# Application of Alcohols to Inhibit the Formation of Ca(II) Dodecyl Sulfate Precipitate in Aqueous Solutions

**DOI:** 10.3390/ma17081806

**Published:** 2024-04-15

**Authors:** Csaba Bús, Marianna Kocsis, Áron Ágoston, Ákos Kukovecz, Zoltán Kónya, Pál Sipos

**Affiliations:** 1 Department of Molecular and Analytical Chemistry, University of Szeged, Dóm Square 7-8, 6720 Szeged, Hungary; bus.csaba@szte.hu (C.B.); mkocsis@chem.u-szeged.hu (M.K.); 2 Department of Physical Chemistry and Material Science, University of Szeged, Rerrich Béla Square 1, 6720 Szeged, Hungary; 3 Department of Applied and Environmental Chemistry, University of Szeged, Rerrich Béla Square 1, 6720 Szeged, Hungarykonya@chem.u-szeged.hu (Z.K.)

**Keywords:** surfactant, precipitation, tensiometry, critical micelle concentration, sodium dodecyl sulfate

## Abstract

The presence of alkaline earth cations, in particular, Ca^2+^ and Mg^2+^ ions in brine, causes undesired effects in solutions containing anionic surfactants because of precipitate formation. In the present study, an anionic surfactant, sodium dodecyl sulfate (SDS), was investigated, focusing on the determination of various properties (surface tension, critical micelle concentration, micelle size, turbidity) in the presence of alcohols and, in particular, the inhibition of the precipitation of SDS with calcium ions. The calcium ions were added to the surfactant in increasing concentrations (3.0–10.0 g/L), and short-carbon-chain alcohols (methanol, ethanol, *n*-propanol and *n*-butanol) were used to shift the onset of precipitate formation. The critical micelle concentration (CMC) of SDS in the presence of alcohols was also determined. It was established that among these alcohols, methanol and ethanol did not exert significant effects on the solubility of the Ca(DS)_2_ precipitate, while *n*-propanol and *n*-butanol were found to be much more efficient inhibitors. In addition, all the alcohols in the applied concentration range (up to 20 V/V%) were found to decrease the critical micelle concentration of SDS.

## 1. Introduction

Long-chain alkyl sulfonates and sulfates are a commonly applied anionic surfactants [1]. The main functions of surfactants include reducing interfacial tension (IFT) and the alteration of wettability. In some cases, the IFT between water and oil can be reduced from 20–30 mN/m to a value in the order of 10^−3^ mN/m as a result of surfactant addition [2], but generally, this decrease is lower (i.e., 10 mN/m [3]). Numerous parameters have been described to affect IFT, e.g., temperature, pressure, salinity, surfactant type and concentration, and solute type and concentration [4,5]. 

Divalent cations, most importantly, alkaline earth metal ions (i.e., Ca(II) and Mg(II)) are known to adversely influence the applicability of surfactants. These cations may be bound to the polar head of the anionic surfactants, which affects surface tension (ST) reduction. In waterflooding precipitation, dynamic conditions need to be considered [6]. Furthermore, anionic surfactants may form precipitates with these divalent cations if the latter are present in sufficient concentrations. For example, precipitation with these divalent cations is not expected in brine water with a divalent cation concentration of less than 15–900 ppm [7,8].

Several studies aimed at the inhibition of such precipitate formation have been published in the open literature. Zhang et al. demonstrated that [B(OH)_4_]^−^ ions are effective complexing agents of bivalent cations, like Ca^2+^ and Mg^2+^, that increase their effective solubility in the aqueous phase. According to their results, at first, an amorphous precipitate is formed which dissolves as the divalent cation concentration is increased [9].

Khaled et al. applied in situ-generated sodium acrylate to inhibit precipitation caused by divalent cations [10]. They claimed that a delicate interplay among three parallel effects is responsible for the efficiency of the inhibition: the direct complexation of sodium acrylate with the ions present in the solution; the adsorption of sodium acrylate on the crystal surface or at the active growth sites of the solid phase present; and a change in the ionic strength of the solution as a result of sodium acrylate addition, and hence, an increase in the effective solubilities of the calcium and magnesium compounds [10]. The advantage of the in situ production of the inhibitor is the use of hard brines without the need for softening the injection water [11].

Tri-sodium citrate was used to sequester calcium in a study published by Miyazaki et al. The experimental data confirmed that tri-sodium citrate prevents the precipitation caused by calcium ions up to 900 ppm in solutions containing surfactin (a class of biosurfactant with an uncommon cyclic lipopeptide as a head group) [12]. 

Different electrolytes were reported to reduce the CMC of both anionic and non-ionic surfactants [13]. The added salts exert a shielding effect in solutions of anionic surfactants, which suppresses the dissociation of the ions from the micelle, resulting in a decrease in the repulsive forces between the polar head groups [14]. 

Alcohols are the most commonly used cosolvents of surfactants to improve their properties. Alcohols were also described as cosurfactants which can stabilize microemulsions in mixtures with oil. Some studies are concerned with the interactions between surfactants and alcohols. The effects of alcohols on CMC depend on the chain length of the alcohols [15]. Alcohols with shorter carbon chains are miscible with water, split between the micelles and the bulk phase [16]. According to Rao et al., in solutions of anionic surfactants the C_4_–C_7_ alcohols decrease the CMC, which is associated with an increase in the distance between the head groups through solubilization and coordination with the micelle surface. Furthermore, the interaction between the alkyl chains of the surfactants and those of the alcohols results in an increase in entropy [17,18]. Methanol and ethanol are reported to increase the CMC value in solutions of polyoxyethylene lauryl ethers according to the facilitation of solvation by water, while alcohols with longer carbon chains (C_3_–C_7_) are reported to be able to intercalate into the micellar structure, forming mixed micelles, which also decreases the CMC [19,20].

The CMC of SDS in aqueous solutions has been determined in a large variety of studies by using various experimental techniques (including dynamic light scattering, fluorescent microscopy, UV-Vis spectroscopy, tensiometry and conductometry). The different experimental techniques provide various CMC values of SDS ranging from 3.46 mmol/L to 8.03 mmol/L. These concentration values are equal to a 1.0–2.32 g/L SDS concentration [21,22,23,24,25,26,27,28].

The present paper is concerned with the effect of alcohols on the surfactant precipitation induced by the addition of calcium ions. The results indicate that under well-defined conditions, alcohols with carbon chains longer than C_2_ are capable of inhibiting precipitation. The inhibition effects of *n*-propanol and *n*-butanol on the calcium ion-induced precipitation in SDS solutions are also presented in this paper. 

## 2. Materials and Methods

Calcium chloride dihydrate (CaCl_2_ × 2H_2_O), methanol, ethanol, *n*-propanol and *n*-butanol were purchased from VWR Chemicals (Debrecen, Hungary). Sodium dodecyl sulfate (C_12_H_25_NaO_4_S, SDS) was purchased from Sigma-Aldrich (St. Louis, MO, USA). All chemicals were used without further purification. All solutions were prepared using MilliQ-MilliPore water, which was produced by reverse osmosis and was further purified by UV irradiation, using a Puranity TU3 UV/UF+ system (VWR, Debrecen, Hungary).

Mass spectrometric measurements were processed with a 1260 Infinity II HPLC setup coupled to a G6125B LC-MSD (mass-selective detection) from Agilent (Santa Clara, CF, USA), applying electrospray ionization (ESI). For analysis, a ~0.075 g/L (75 ppm) aqueous surfactant solution was prepared, and ultrapure water was used as an eluent. The mass spectrometric measurements were carried out in negative-ion mode, scanning the *m*/*z* region from 150 to 400.

**Figure 1:**
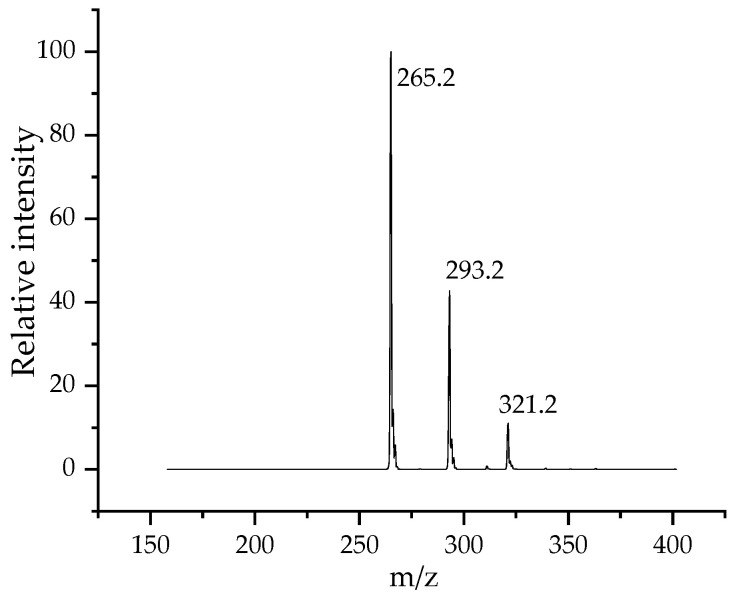
Mass spectrum of the as-received sodium dodecyl sulfate.

The exact composition of the SDS was determined by using LC-MS. The mass spectrum of the SDS is shown in Figure 1. 

The molar mass of the dodecyl sulfate (DS^−^) anion was 265.2 g/mol. The recorded mass spectra indicated the DS^−^ anion to be the main surfactant compound, but two other surfactant homologues were also detected; the 293.2 *m*/*z* value indicated a tetradecil sulfate, while the third compound was a hexadecil sulfate with a value of 321.2 *m*/*z*. The relative intensity values showed that the investigated surfactant was a mixture of the C_12_ homologue in a proportion of 67% (*w*/*w*), C_14_ at 27% (*w*/*w*), and C_16_ at 6% (*w*/*w*), respectively. From these data, an average molar mass of 299.12 was calculated and used to calculate the molar concentration of SDS.

The turbidity values were measured by using an HI-98703 Precision Turbidity Portable Meter (Hanna Instruments, Keysborough, Australia). The instrument was calibrated according to the calibration standards provided by the supplier (0.1, 15, 100 and 750 NTU). The reported results are based on three independent measurements. 

The surface tensions at the air–water interface and the CMC values of the surfactant solutions were determined at 25.0 ± 0.1 °C using a tensiometer (type K100; Krüss, Hamburg, Germany). The measurements were carried out using a Wilhelmy plate. 

Dynamic light scattering (DLS) measurements were carried out by using a Malvern Zetasizer Nano ZS instrument (Malvern, UK) operating with a 4 mW helium–neon laser light source (λ = 633 nm) for the determination of micelle size values. The measurements were carried out at room temperature using quartz cuvettes. The measurements were made in back-scattering mode at an incident angle of 173°. At the start of the measurement, a 120 s equilibration time was employed. The reported data were calculated from three independent measurements. The data evaluation was carried out by using the Zetasizer software (7.11). To minimize the effect of the presence of solid contaminants (for example, airborne dust), the solutions were filtered through a 0.1 μm diameter filter before measurements. The refractive index of the solvents were measured by a Mettler Toledo RM50 refractometer (Mettler Toledo, Columbus, OH, USA) at room temperature, and the viscosity values were measured using and Ostwald-type viscosimeter (VWR Chemicals, Debrecen, Hungary) at room temperature (Supporting information, Table S1). 

For the measurements, SDS solutions with 0.1, 0.5, 1.0, 2.5 and 5.0 g/L concentrations were prepared using MilliQ water. The effects of the investigated alcohols on the SDS solutions were also studied; to the aqueous SDS solutions, 5.0 V/V%, 10.0 V/V% and 20.0 V/V% methanol, ethanol or *n*-propanol were added. In the case of *n*-butanol, the maximum amount of alcohol was 5.0 V/V% because at higher concentrations, *n*-butanol was found to be immiscible with the aqueous surfactant solutions.

From calcium chloride dehydrate, stock solutions with concentrations of 3.0, 5.0 and 10.0 g/L (with respect to calcium ions) were prepared using MilliQ water. The effects of calcium ions on the SDS solutions were determined by using the solution with the highest (5.0 g/L) SDS concentration. The calcium ions were then added to the SDS-containing samples in the form of an aqueous solution. Upon adding alcohols to the samples, their concentrations were made up to 10.0 V/V% (except for *n*-butanol; see above); here, we attempted to take the high environmental load of this process into consideration [29]. For samples where the precipitate was visibly not dissolved, the amount of alcohol was increased to 20.0 V/V%. 

During the experiments, the alcohols were added to the surfactant solutions first, before adding the stock solutions containing Ca^2+^. 

## 3. Results and Discussion

### 3.1. SDS in Various Alcohol–Water Mixtures

#### 3.1.1. Determination of CMC of SDS in Alcohol–Water Mixtures

**Figure 2:**
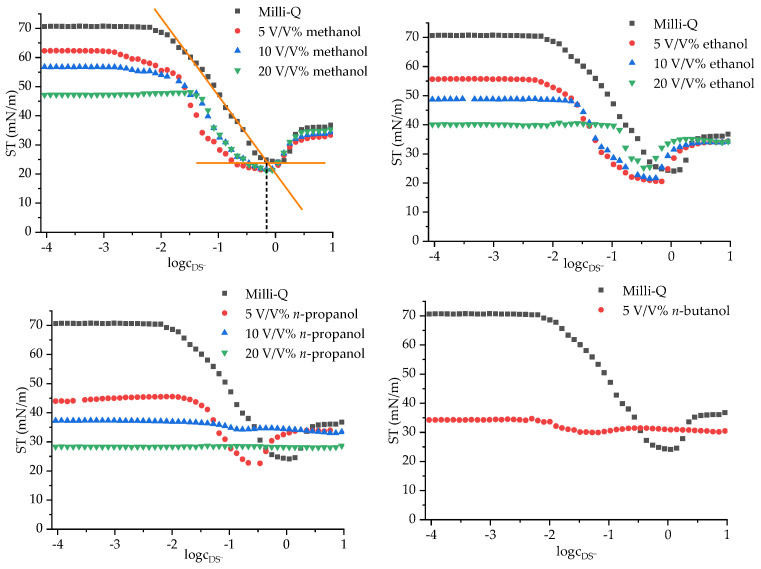
Tensiometric curves of the aqueous and alcohol-containing SDS solutions (c_DS^−^_ expressed in g/L).

The CMC values were determined via surface tension measurements. To the SDS solutions, 5.0, 10.0 and 20.0 V/V% alcohol–water mixture was added as a titrant. The titrated stock solution was prepared with a starting surfactant content of 10.0 g/L. To determine the CMC value, the measured surface tension values were plotted as a function of the logarithm of the surfactant concentration. The titration curve of the aqueous and alcohol-containing SDS solutions are illustrated in Figure 2.

The critical micelle concentrations obtained from the tensiometric curves are listed in Table 1. The way the CMC was extracted from the curves shown in Figure 1 is demonstrated in the upper left figure. For CMC, the lower intersection point was always chosen; there is another section on the titration curves where the ST becomes constant; according to the literature [30], the second breakpoint at higher SDS concentrations is likely to correspond to a second CMC, with micelles exhibiting structures different from those corresponding to the first one.

On the basis of the tensiometric measurements, the aqueous SDS solution has a somewhat lower CMC than the values reported in the literature; this may be associated with the way of extracting the CMC from the tensiometric curve. Short-carbon-chain-length alcohols (methanol and ethanol) were reported to increase the CMC of SDS [16,31] relative to the CMC in pure water. In our case, the addition of any alcohol resulted in CMC values which increase with increasing alcohol concentration but are always smaller than that in pure water, even at the largest alcohol concentrations. The reason for this most probably lies in the alcohol concentrations being much larger in our systems than in those reported in the literature. The addition of propanol had considerable effects on the tensiometric behavior of SDS: in the presence of 5.0 V/V% and 10.0 V/V% propanol, the CMC decreased, but in the presence of 20.0 V/V%, the titration curve did not have an inflexion point; thus, CMC determination was not possible. This curve indicates that higher *n*-propanol content inhibits micelle formation. The CMC values measured in *n*-propanol-containing solvents are consistent with the literature values [31]. The addition of 5.0 V/V% *n*-butanol considerably decreased the CMC from 0.66 g/L to 0.04 g/L. These results were also in line with data published in the literature [31]. 

#### 3.1.2. Measurement of Micelle Size Values in the Presence of Alcohols

Dynamic light scattering (DLS) is a conventional technique used to measure micelle size values [32,33]. For the DLS measurements in this study, SDS solutions were prepared in a broad concentration range both below and over the critical micelle concentration determined via tensiometry. The alcohol-containing solutions were prepared with the same composition as the aqueous ones.

SDS was previously reported to form micelles in water with diameters between 3.5 and 4.0 nm [34,35]. The micelle size values obtained by us are listed in Table 2.

The obtained micelle diameter values do not indicate micelle formation below a 1.0 g/L SDS concentrations. Under a 1.0 g/L surfactant concentration, larger aggregates were extracted from the experimental data; they are supposedly fitting artefacts which are associated with the small number of inhomogeneities remaining in the solution (in spite of the careful filtration). Hereafter, micelle diameters over 100 nm should be considered artefacts as well (Supporting Information, Figures S1–S29). In solutions with 1.0–5.0 g/l surfactant concentrations, micelles with 3–7 nm diameters were detected, which corresponds to the micelle size found in the literature [34,35]. 

The addition of alcohols did not considerably change the micelle size. The corresponding data are shown in Table 3. 

During the dissolution of the surfactant in a medium containing methanol or ethanol, a higher alcohol fraction remains in the intermicellar phase and a smaller part is solubilized [16]. Alcohols with carbon chains below C_3_ were reported to decrease micelle size [36]. Our experimental results confirmed the CMC-decreasing effect of alcohols obtained from the tensiometric measurements. The micelle-size-decreasing effects of methanol and ethanol were confirmed by these results until 10.0 V/V% alcohol content was reached. However, in the presence of 20.0 V/V% ethanol-containing media, the micelle size values did not follow the same trends; with an increase in the surfactant concentration, the micelle size values increased. The micelle size values determined in *n*-propanol and *n*-butanol containing media are shown in Table 4.

The results obtained for *n*-propanol- and *n*-butanol-containing solutions follows trends similar to those obtained for methanol. Increasing the surfactant concentration did not decrease the micelle size until 1.0 g/L-containing SDS solutions were reached; moreover, the CMC of SDS was found to be lower than 0.5 g/L in the presence of *n*-propanol. The values recorded in 20.0 V/V% *n*-propanol indicate the inhibition of micelle formation (Supporting Information Figures S30–S41), or even the dissolution of the micelles. These results confirm the results extracted from the tensiometric curves. 

*n*-butanol was determined to be highly solubilized in the micelles and to cause a rapid decrease in micelle size [37]. Our results did not indicate this considerable decrease until a 2.5 g/L surfactant concentration. Moreover, in the case of *n*-butanol, all the studied samples contained SDS in concentrations above the CMC. According to the observed particle size values recorded in *n*-butanol-containing media and comparing these with the tensiometric results, it is indicated that in the presence of *n*-butanol, a molecularly dispersed solution is formed instead of a colloid solution with a micellar structure (Supporting Information Figures S42–S46). 

### 3.2. Inhibition Effect of Alcohols on the Precipitation of DS^−^ with Calcium Ions 

#### 3.2.1. Determination the Inhibition Effects of Alcohols on the Precipitation Using Turbidimetry 

The precipitation of DS^−^ with calcium ions was investigated in solutions containing 5.0 g/L SDS. During the first measurement, the calcium ion concentration was varied from 3.0 g/L to 10.0 g/L. First, the alcohols were added to the samples in a 10.0 V/V% concentration (in the case of *n*-butanol, 5.0 V/V%). In samples where large amounts of precipitate occurred (ethanol and methanol), the amounts of the added alcohols were increased to 20.0 V/V%. The precipitation was characterized by using turbidimetry, which is an effective method to make distinctions between transparent and precipitated solutions.

**Figure 3:**
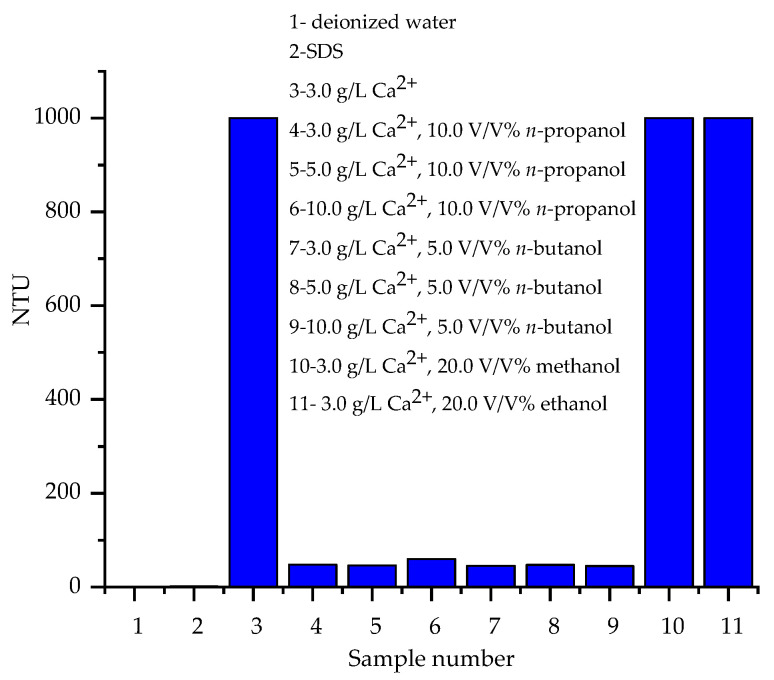
Turbidity values (in arbitrary unit) of Ca^2+^-containing SDS solutions (c_SDS_ = 5.0 g/L, c_Ca_2_^+^_ = 3.0–10.0 g/L) in the presence of various alcohols.

The turbidity values of the calcium- and alcohol-containing samples are shown in Figure 3.

The turbidity values approximately represent the inhibition effects of alcohols on the precipitation of DS^−^ with Ca^2+^ ions. The addition of 10.0 V/V% *n*-propanol enhanced the solubility of Ca(DS)_2_ precipitates and successfully inhibited precipitation at all investigated Ca^2+^ concentrations. The addition of 5.0 V/V% *n*-butanol to the calcium ion-containing SDS solutions resulted in similar turbidity values compared to the *n*-propanol-containing ones, these turbidity values confirmed the inhibition effects of *n*-butanol on calcium ion-induced precipitation in SDS solutions. In the presence of 20.0 V/V% methanol and ethanol, considerably higher (˃1000) NTU values were measured. Such high turbidity values showed the presence of high amounts of solid precipitates in the samples. Therefore, methanol and ethanol were found to have no considerable effect on the solubility of the precipitates, even at the highest 20.0 V/V% concentration.

The inhibition effects of *n*-propanol and *n*-butanol were further studied by increasing the calcium ion concentration in the samples. Solutions with 10.0 V/V% *n*-propanol and 5.0 V/V% *n*-butanol containing 5.0 g/L SDS were prepared by adding the calcium ion-containing stock solutions. Of these solutions, the *n*-propanol-containing ones were found to be “calcium resistant” until the above-mentioned 10.0 g/L Ca^2+^ concentration was reached, while the *n*-butanol-containing samples remained transparent even after reaching a 100.0 g/L Ca^2+^ concentration. Although the metal ion concentration was not increased above this concentration, this experiment indicated that the addition of *n*-butanol results in the collapse of the SDS’s micellar structure and therefore inhibits the precipitation of Ca(DS)_2_.

The methanol- and ethanol-containing solvents were also further investigated to determine the minimum amount of calcium which induces precipitation in the presence of these alcohols. During the experiments, 5.0 g/L SDS stock solutions with 10.0 V/V% and 20.0 V/V% methanol and ethanol were prepared. A total of 6.0 mL of the alcohol-containing solution was added to the sample holder of the turbidity meter. Ca^2+^ titrant solution (1.5 g/L) was added to the surfactant solution in 100 μL increments and the turbidity was measured after each step. The calcium solution was added to the alcohol-containing solution until precipitation was detected, and the surfactant–calcium ion molar ratios were calculated. 

**Figure 4:**
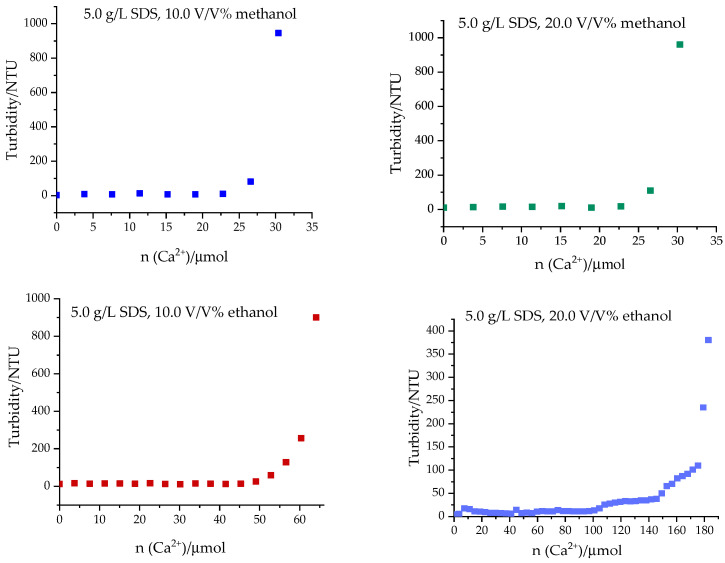
Turbidity of the methanol- and ethanol-containing SDS solutions as a function of the amount of added Ca(II).

The experimental results are illustrated in Figure 4. 

The sudden increase in the turbidity values indicates the onset of the formation of precipitates. From the volume and concentration of the calcium solution, the corresponding molar ratios were calculated. The measurements indicated that the solutions containing methanol become turbid at an SDS/Ca^2+^ = 4.67 molar ratio, independently of the amount of methanol present. In ethanol–water mixtures, this value was found to be SDS/Ca^2+^ = 2.06 at 10.0 V/V% and 0.68 at 20.0 V/V%. Accordingly, in comparison, the Ca(DS)_2_ precipitate is more soluble in methanol–water mixtures than in ethanol–water ones.

#### 3.2.2. Measurement of Micelle Diameters in SDS Solutions Containing Calcium and Alcohol

**Figure 5:**
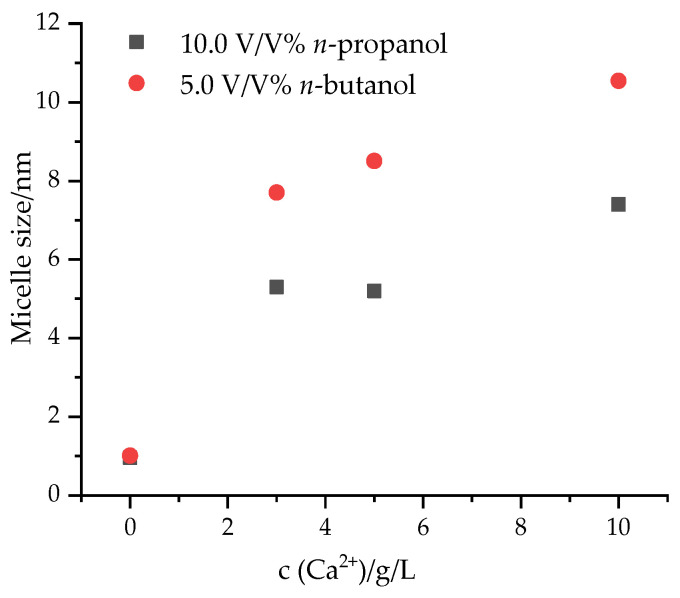
Micelle size values of calcium ion-containing SDS solutions in the presence of alcohols (c_SDS_ = 5.0 g/L).

As *n*-propanol and *n*-butanol did, while methanol and ethanol did not, enhance the solubility of Ca(DS)_2_ precipitates, the micelle size values were determined only for the *n*-propanol- and *n*-butanol-containing solutions. The micelle size values of Ca^2+^-containing samples in the presence of these alcohols are shown in Figure 5.

In the presence of 10.0 V/V% *n*-propanol and 5.0 V/V% *n*-butanol, SDS in a 5.0 g/L concentration was found to be present in molecular form (0.96 nm diameter in 10 V/V% *n*-propanol solution and 1.01 nm diameter in 5 V/V% *n*-butanol). With the addition of calcium to these systems, micelles are indeed formed. The micelle size values increased upon increasing the metal ion concentration in the presence of both alcohols. In *n*-propanol-containing media approximately 5.5–7.0 nm micelle sizes were detected. The presence of *n*-butanol resulted in the formation of larger micelles measuring 8.0–11.0 nm diameter. According to these experimental results, it can be concluded that higher micelle sizes facilitate the solubilization of SDS in the presence of calcium ions.

#### 3.2.3. Measurement of Surface Tension Samples of Calcium- and Alcohol-Containing SDS Samples

**Figure 6:**
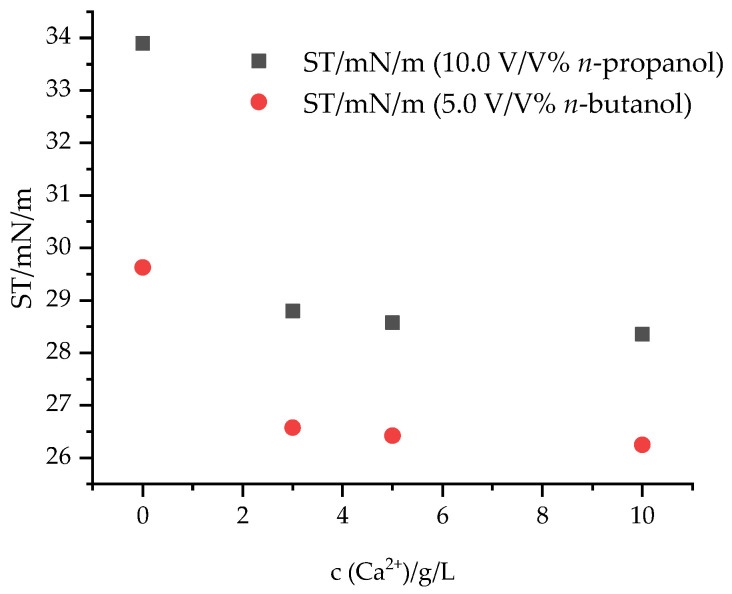
Surface tension of calcium ion- and alcohol-containing SDS solutions (c_SDS_ = 5.0 g/L).

The surface tension was measured in calcium ion-containing SDS samples in the presence of *n*-propanol and *n*-butanol. The results are presented in Figure 6.

Generally, the addition of alcohols resulted in a decrease in the surface tension of solutions containing SDS and calcium ions. Upon comparing the alcohol-containing samples, it can be stated that in the presence of *n*-propanol, higher surface tensions were measured; therefore, the larger the carbon chain length, the larger the decrease in the surface tension both in the presence and in the absence of calcium ions.

## 4. Conclusions

In the present study, the effects of alcohols on the inhibition of the precipitation of sodium dodecyl sulfate (SDS) with calcium ions were studied. The samples were characterized by using tensiometry, turbidimetry and dynamic light scattering. All the investigated alcohols (methanol, ethanol and *n*-propanol) decreased the CMC of the SDS-containing solutions (depending on the type and added amount of alcohol) from 0.66 g/L to 0.148–0.346 g/L, while in the presence of 5.0 V/V% *n*-butanol, 0.03 g/L CMC was obtained. Among the alcohols, *n*-propanol and *n*-butanol inhibited the formation of Ca(DS)_2_ precipitation in 5.0 g/L SDS solutions until a 10.0 g/L Ca^2+^ concentration was reached. The addition of calcium ions increased the micelle size values to 7.4 nm in *n*-propanol- and to 10.5 nm in *n*-butanol-containing solutions.

In the presence of 10.0 V/V% propanol and butanol, the calcium ion concentration could be increased above 10.0 g/L without the formation of Ca(DS)_2_ precipitates. In the presence of *n*-propanol, the solutions were precipitated when the calcium ion concentration surpassed 10.0 g/L, while the *n*-butanol-containing ones remained transparent even at 100 g/L. This phenomenon strongly indicates the dissolution of micelles under these specific experimental conditions.

According to these results, the use of alcohols as cosolvents may inhibit precipitation caused by divalent ions in SDS solutions. Therefore, the addition of alcohols can be an appropriate method to achieve such inhibition; however, the applicable types of alcohols are limited. The inhibition effects of *n*-propanol and *n*-butanol on calcium ion-induced precipitation were described for the first time in this paper.

## Figures and Tables

**Table 1:** Critical micelle concentrations (CMC) of SDS in the presence of alcohols.

Solvent				
Alcohol V/V%	Methanol	Ethanol	*n*-Propanol	*n*-Butanol
0	0.63 g/L (2.4 mM)			
5.0	0.17 g/L (0.7 mM)	0.16 g/L (0.6 mM)	0.17 g/L (0.7 mM)	0.03 g/L (0.1 mM)
10.0	0.34 g/L (1.3 mM)	0.21 g/L (0.8 M)	0.14 g/L (0.5 mM)	– –
20.0	0.35 g/L (1.3 mM)	0.29 g/L (1.1 mM)	–	

**Table 2:** Micelle size values of SDS in aqueous solutions, obtained from DLS measurements (values marked by an asterisk are supposedly artefacts).

Sample Composition	Particle Size/nm	Polydispersity Index (PdI)
0.1 g/L SDS	167 *	0.3
0.5 g/L SDS	181 *	0.2
1.0 g/L SDS	6.6	0.5
2.5 g/L SDS	4.6	0.5
5.0 g/L SDS	2.9	0.5

**Table 3:** Micelle size and polydispersity index values (in parentheses) of SDS in the presence of methanol and ethanol.

			Solvent			
c (SDS)/g/L	5.0 V/V% Methanol	10.0 V/V% Methanol	20.0 V/V% Methanol	5.0 V/V% Ethanol	10.0 V/V% Ethanol	20.0 V/V% Ethanol
0.5	123.4 (0.2)	17.8 (0.2)	24.4 (0.2)	191.4 (0.4)	83.8 (0.21)	21.8 (0.05)
1.0	4.9 (0.3)	5.9 (0.4)	6.5 (0.5)	5.0 (0.6)	4.5 (0.49)	3.6 (0.10)
2.5	3.5 (0.4)	3.7 (0.3)	18.0 (0.2)	3.0 (0.4)	2.7 (0.35)	58.0 (0.7)
5.0	2.3 (0.6)	2.3 (0.5)	2.0 (0.1)	2.0 (0.4)	1.7 (0.23)	89.3 (0.5)

**Table 4:** Micelle size and polydispersity index values of SDS in the presence of *n*-propanol and *n*-butanol.

Solvent				
c (g/L)	5.0 V/V% *n*-Propanol	10.0 V/V% *n*-Propanol	20.0 V/V% *n*-Propanol	5.0 V/V% *n*-Butanol
0.1	139.9 (0.7)	149.0 (0.6)	156.6 (0.3)	0.70 (0.8)
0.5	51.0 (0.5)	25.6 (0.5)	56.5 (0.8)	0.9 (0.5)
1.0	3.1 (0.5)	53.8 (0.5)	23.2 (0.6)	1.4 (0.42)
2.5	1.7 (0.4)	1.5 (0.7)	1.5 (0.6)	1.3 (0.4)
5.0	1.2 (0.3)	1.0 (0.4)	0.9 (0.4)	1.0 (0.3)

## Data Availability

Data are contained within the article.

## Supplementary Materials

The following supporting information can be downloaded at: https://www.mdpi.com/article/10.3390/ma17081806/s1. Table S1. Viscosity (cP) and refractive index of the applied alcohol-water solvents, Figures S1–S46. Correlation diagrams of dynamic light measurements processed in alcohol containing media.
