# Supplementary material for: Application of Alcohols to Inhibit the Formation of Ca(II) Dodecyl Sulfate Precipitate in Aqueous Solutions
Source: Materials (Basel). 2024 Apr 15;17(8):1806. doi: 10.3390/ma17081806 (PMC11051115; doi:10.3390/ma17081806)
Supplement: Supplementary file 1 [file materials-17-01806-s001.zip › materials-2914036-supplementary.pdf]

Application of Alcohols to Inhibit the Formation of Ca(II) Dodecyl Sulfate Precipitate in Aqueous Solutions

Supporting information

S1

Table S1. Viscosity (cP) and refractive index of the applied alcohol–water solvents

Solvent	Refractive index	Viscosity (cP)
water	1.330	0.8872
5.0 V/V% MeOH	1.336	1.163
10 V/V% MeOH	1.3372	1.243
20 V/V% MeOH	1.3385	1.368
5.0 V/V% EtOH	1.334	1.2
10 V/V% EtOH	1.3405	1.265
20 V/V% EtOH	1.3462	1.639
5.0 V/V% *n*-propanol	1.34	1.32
10 V/V% *n*-propanol	1.3425	1.324
20 V/V% *n*-propanol	1.3449	1.522
5.0 V/V% *n*-butanol	1.3392	1.059

S2

Correlation diagrams of dynamic light scattering measurements processed in alcohol containing media

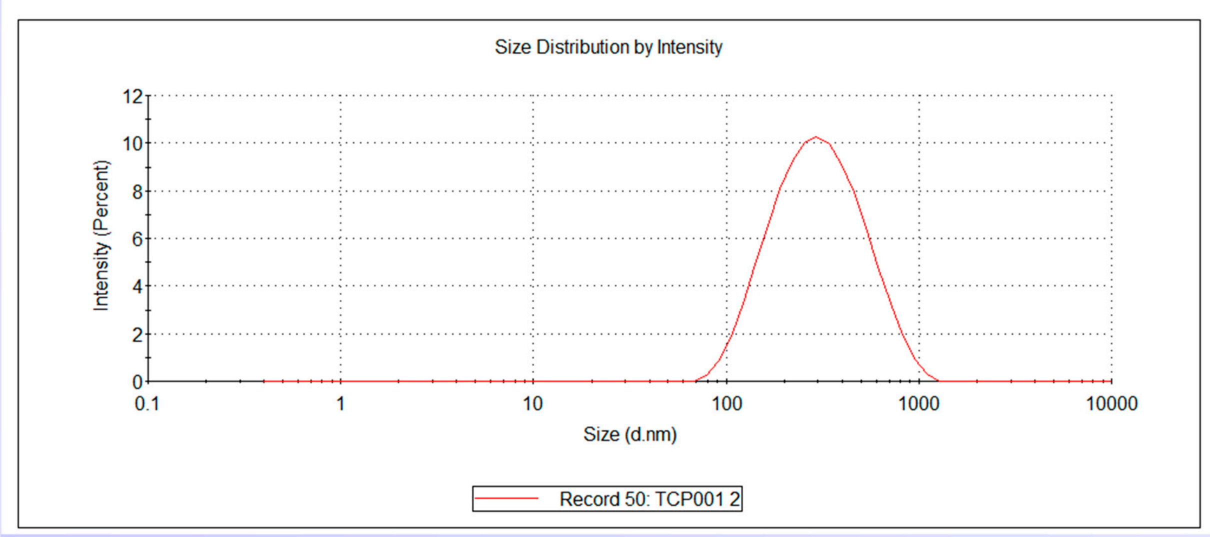

Figure S1. 0.1 g/L SDS

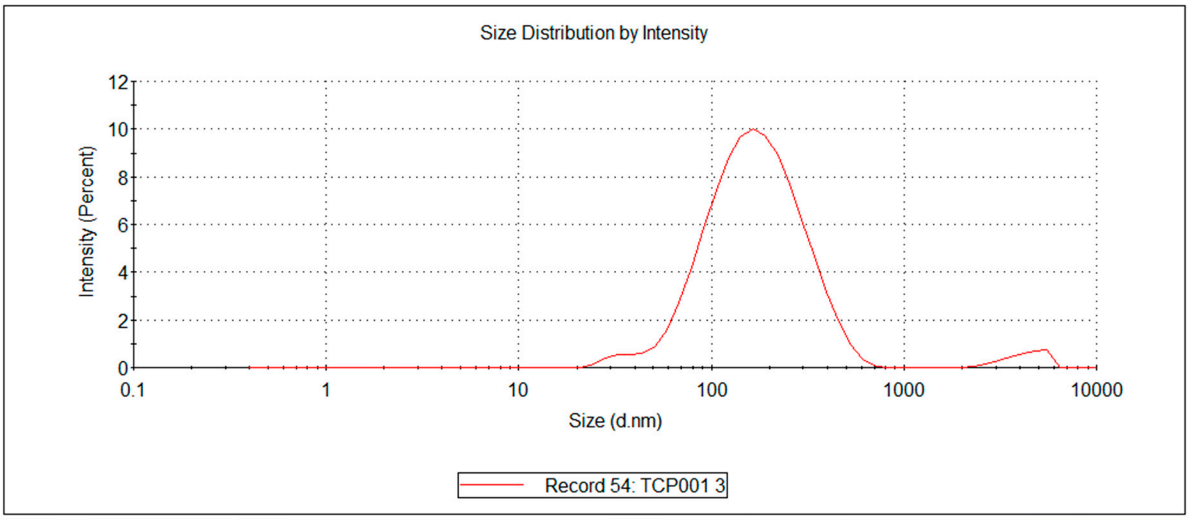

Figure S2. 0.5 g/L SDS

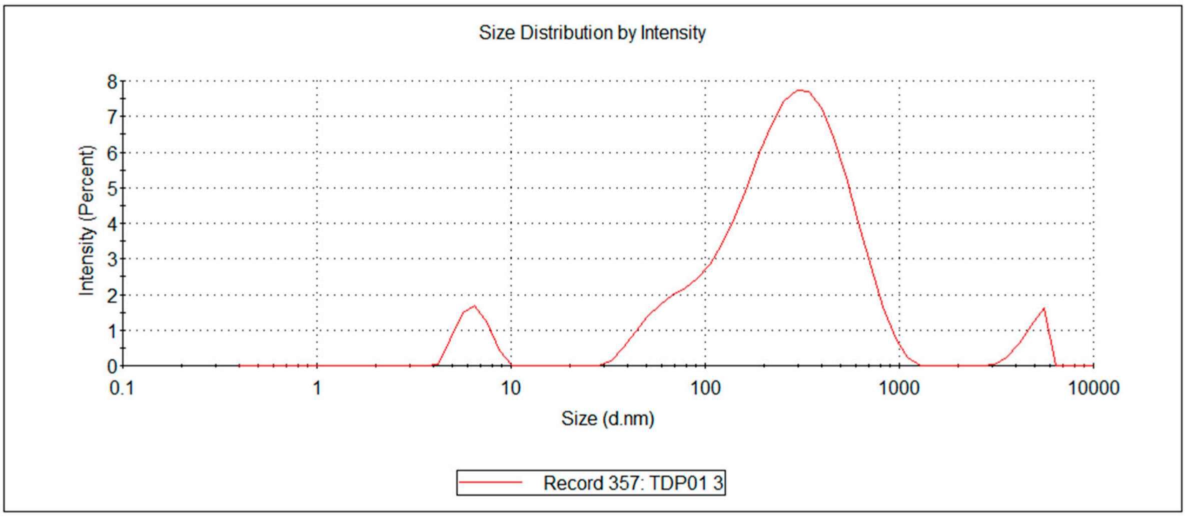

Figure S3. 1.0 g/L SDS

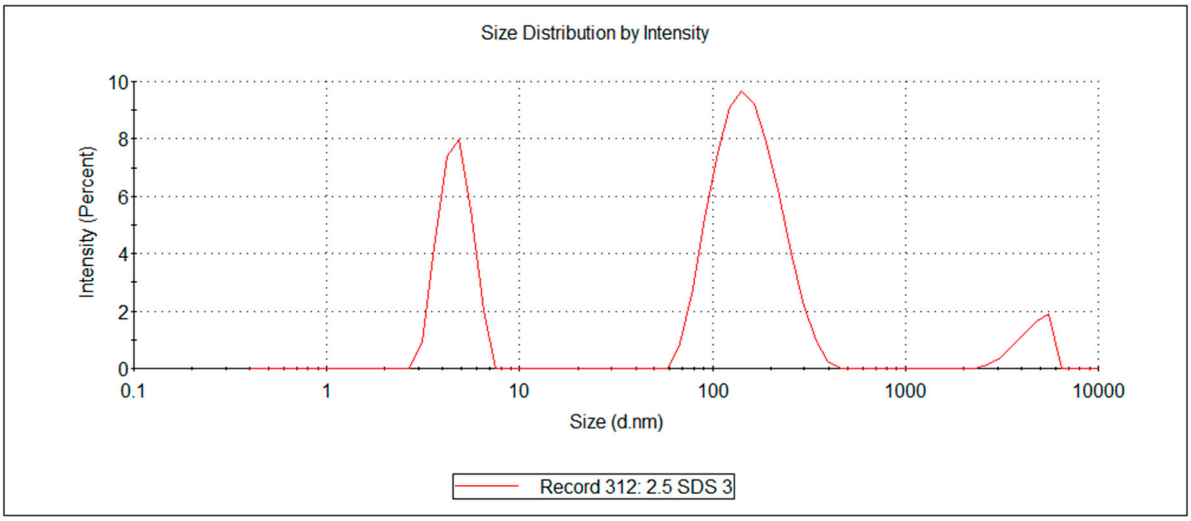

Figure S4. 2.5 g/L SDS

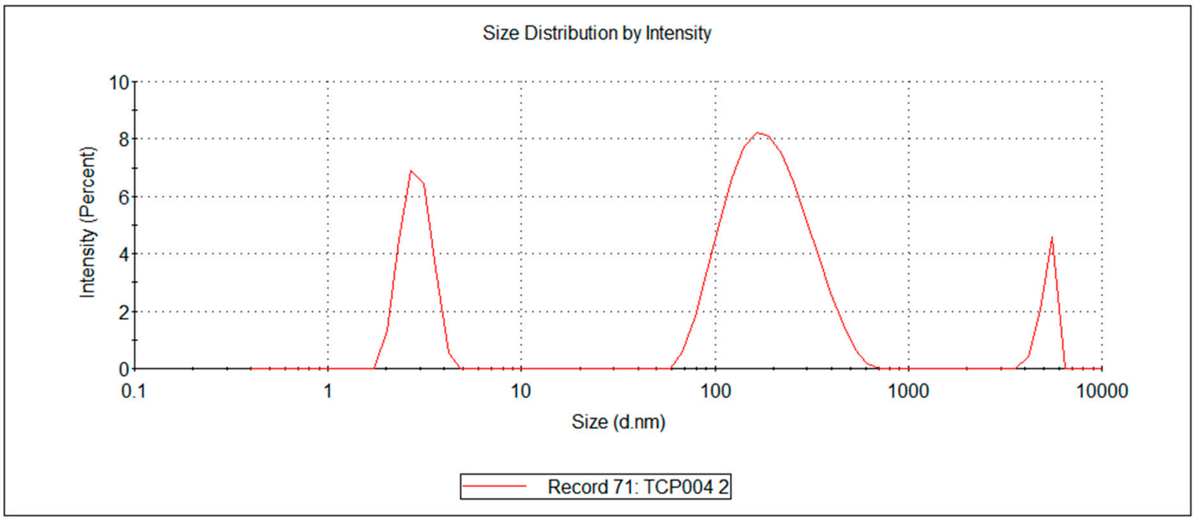

Figure S5. 5.0 g/L SDS

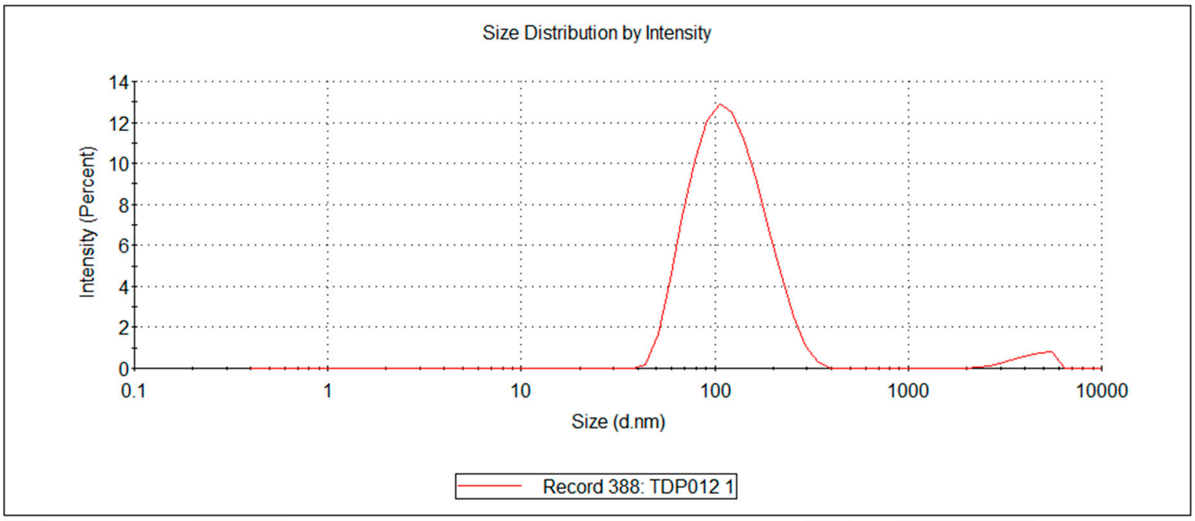

Figure S6 0.5 g/L SDS, 5.0 V/V% MeOH

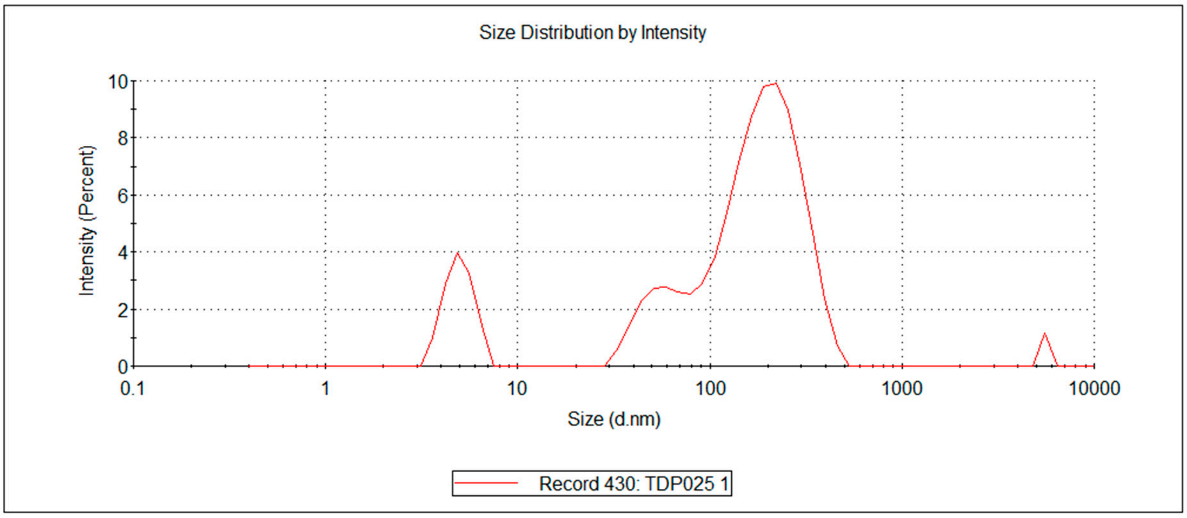

Figure S7. 1.0 g/L SDS, 5.0 V/V% MeOH

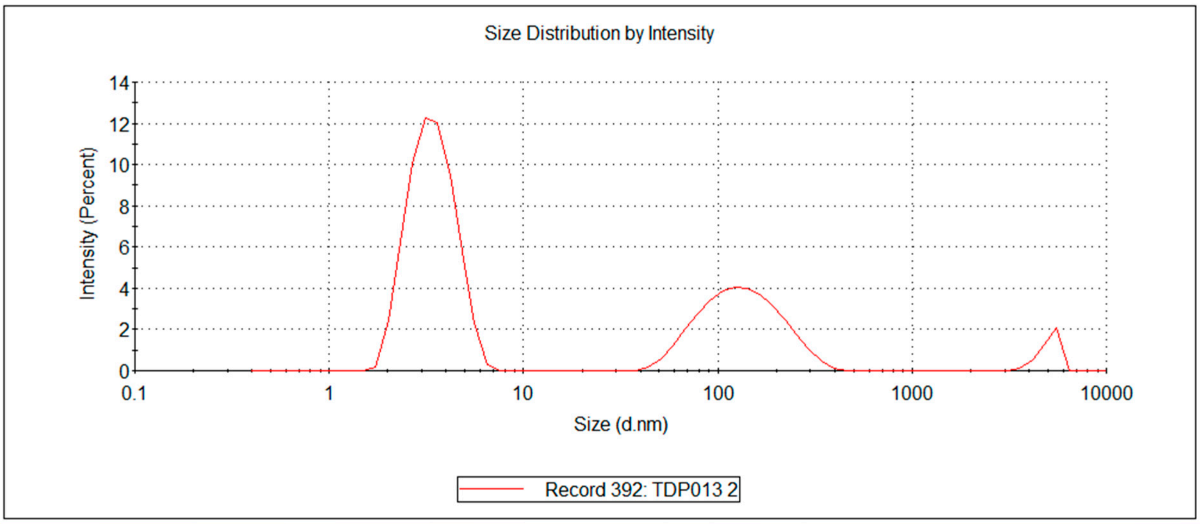

Figure S8. 2.5 g/L SDS, 5.0 V/V% MeOH

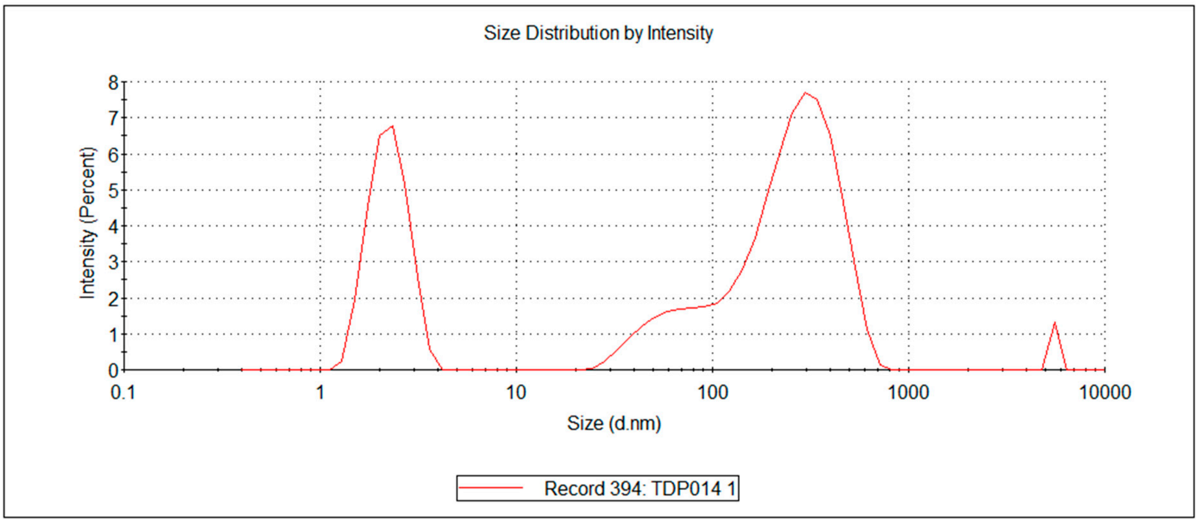

Figure S9. 5.0 g/L SDS, 5.0 V/V% MeOH

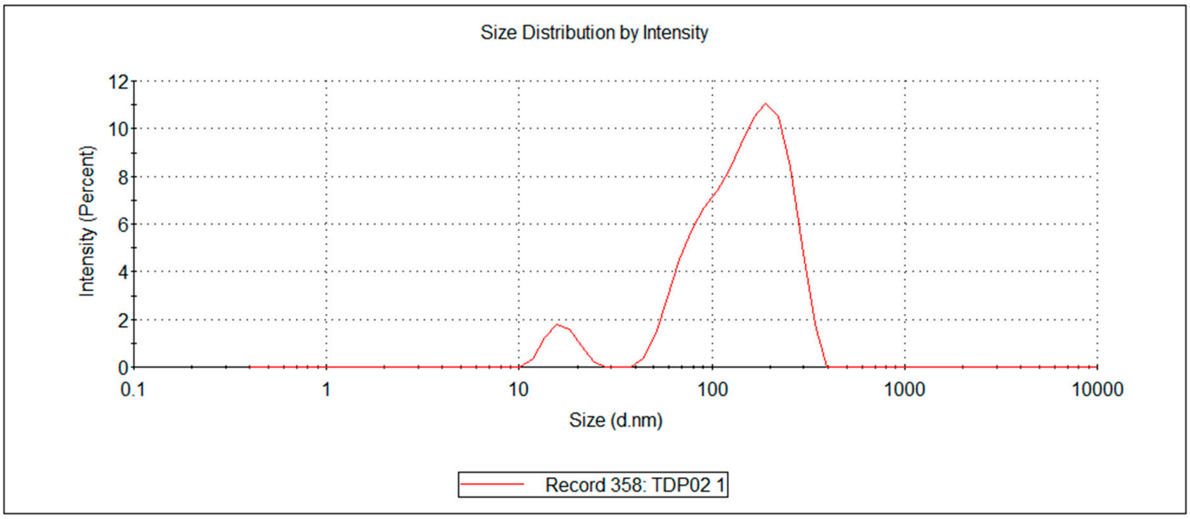

Figure S10. 0.5 g/L SDS, 5.0 V/V% MeOH

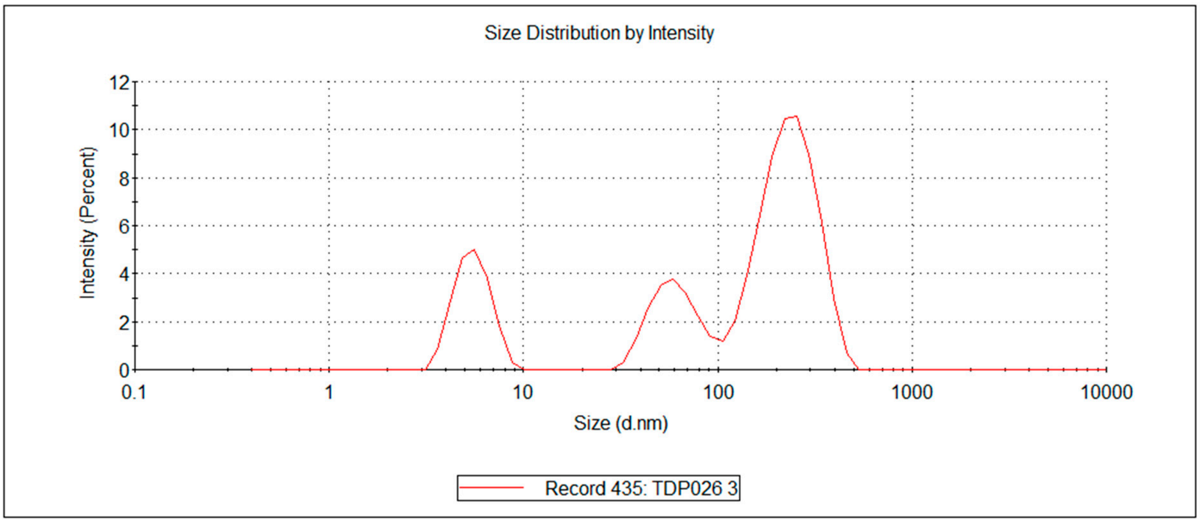

Figure S11. 1.0 g/L SDS, 10.0 V/V% MeOH

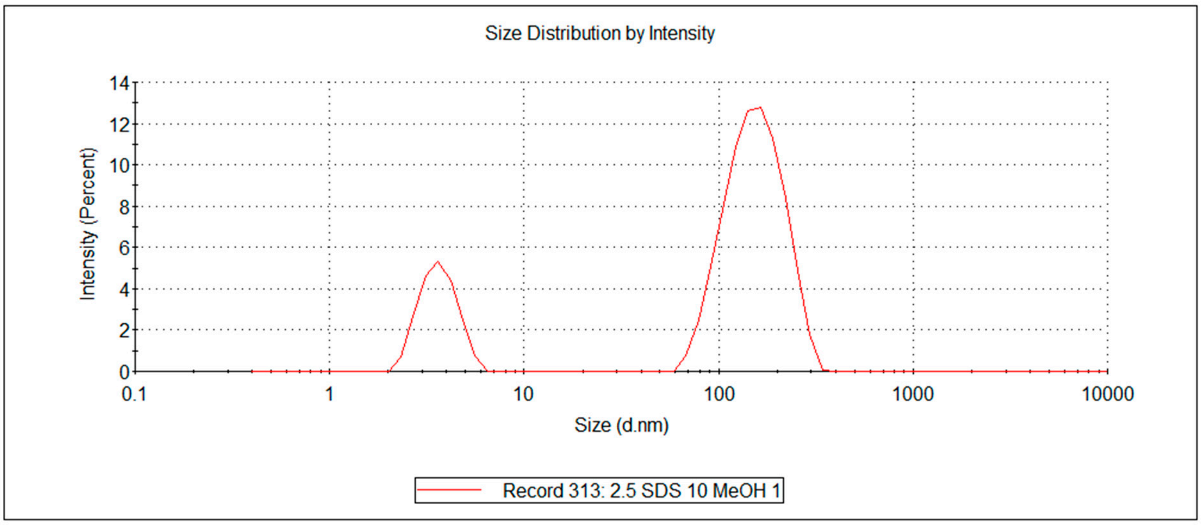

Figure S12. 2.5 g/L SDS, 10.0 V/V% MeOH

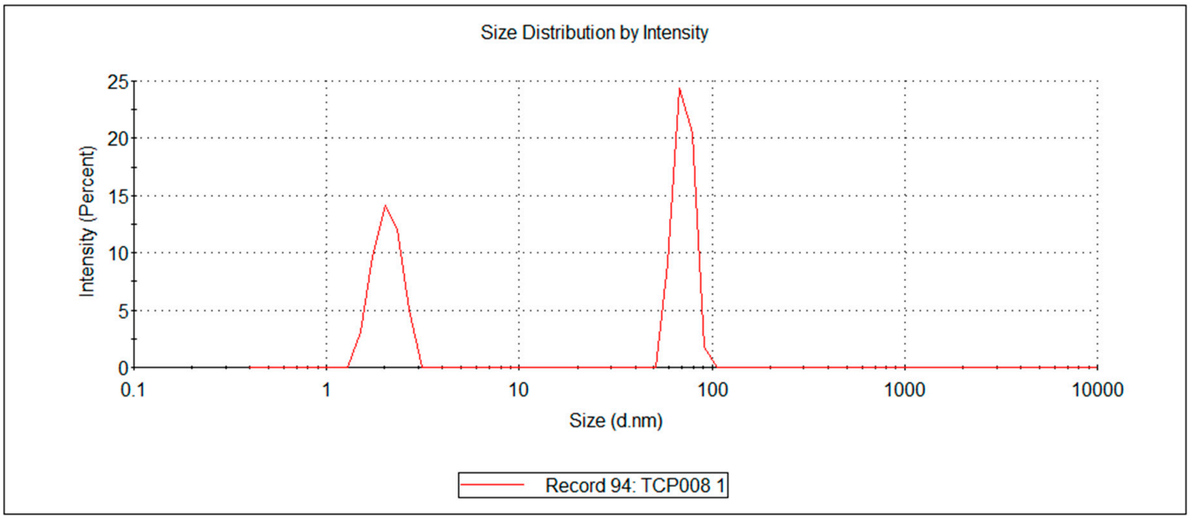

Figure S13. 5.0 g/L SDS, 10.0 V/V% MeOH

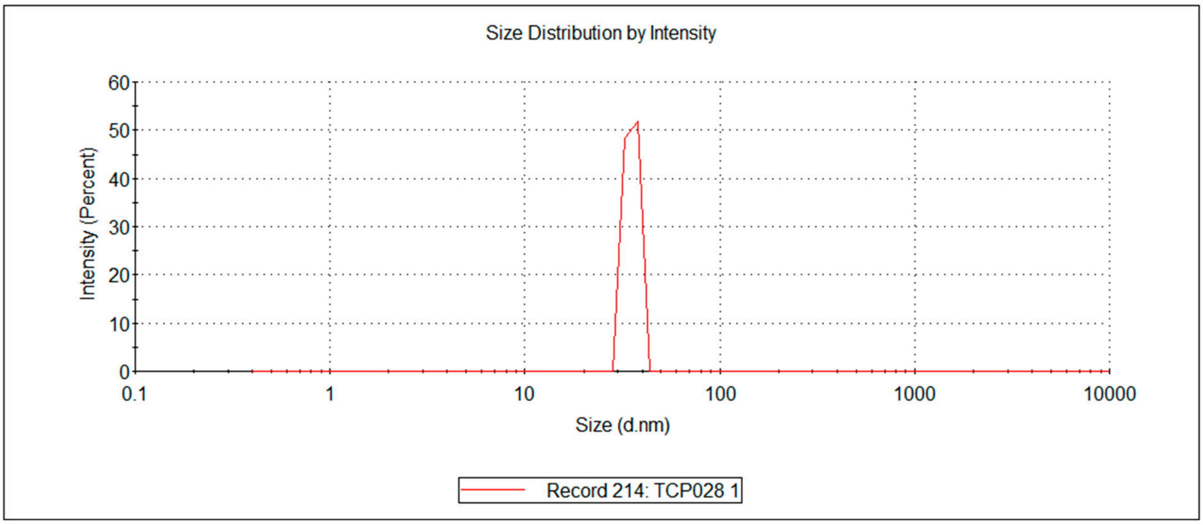

Figure S14. 0.5 g/L SDS, 20.0 V/V% MeOH

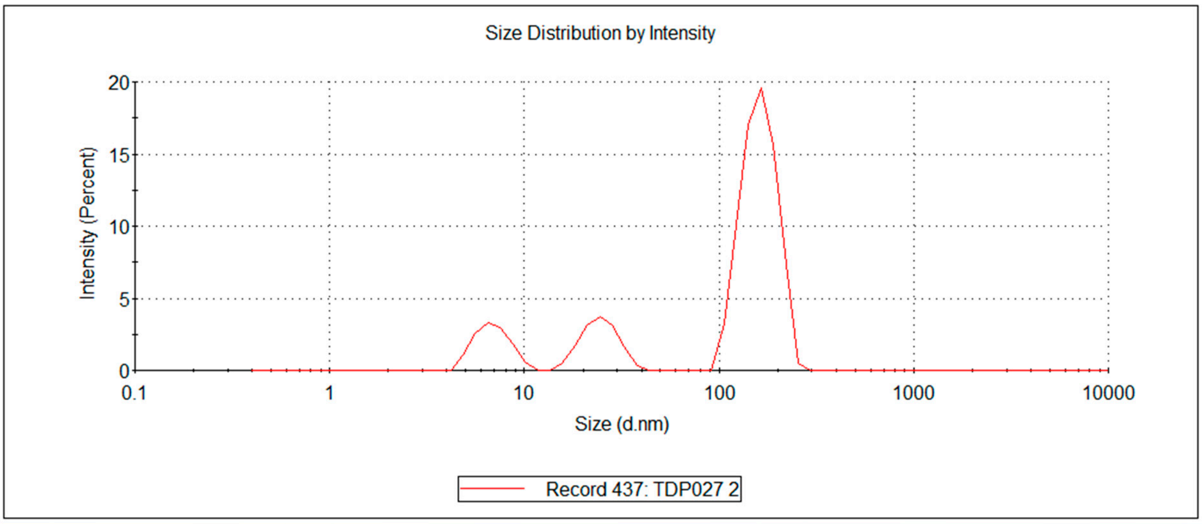

Figure S15. 1.0 g/L SDS, 20.0 V/V% MeOH

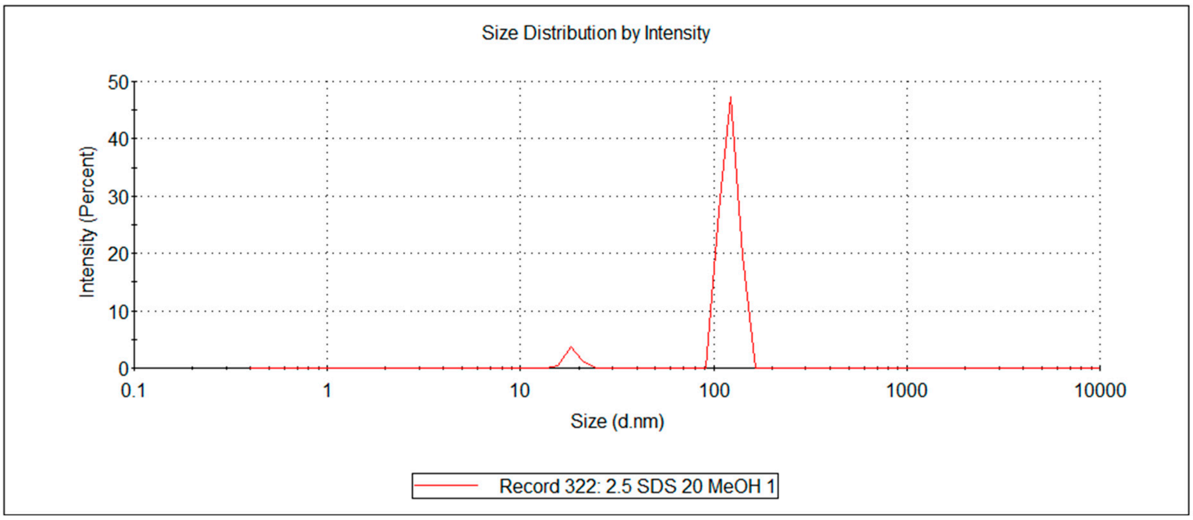

Figure S16. 2.5 g/L SDS, 20.0 V/V% MeOH

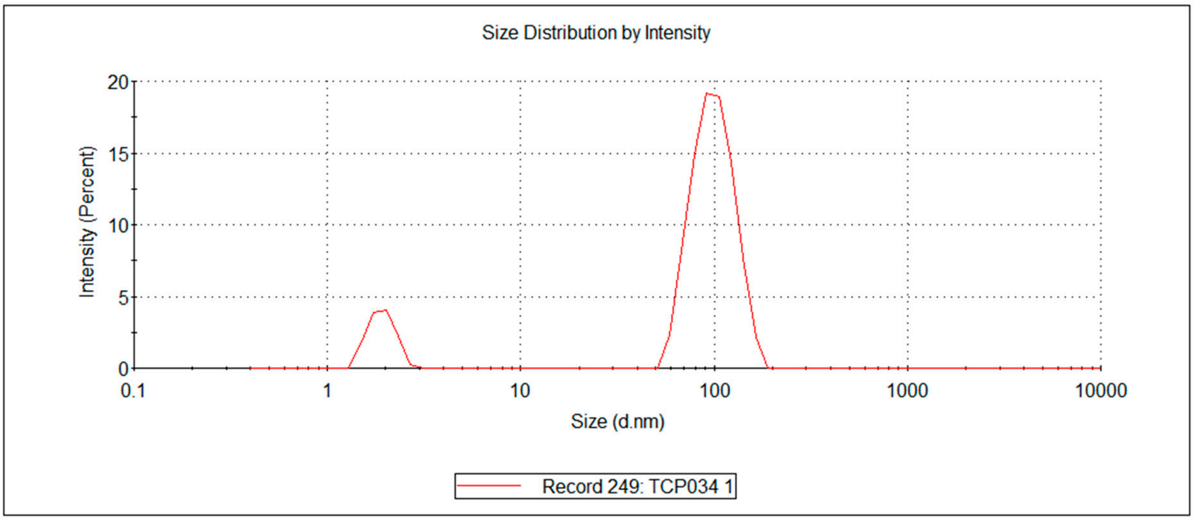

Figure S17. 5.0 g/L SDS, 20.0 V/V% MeOH

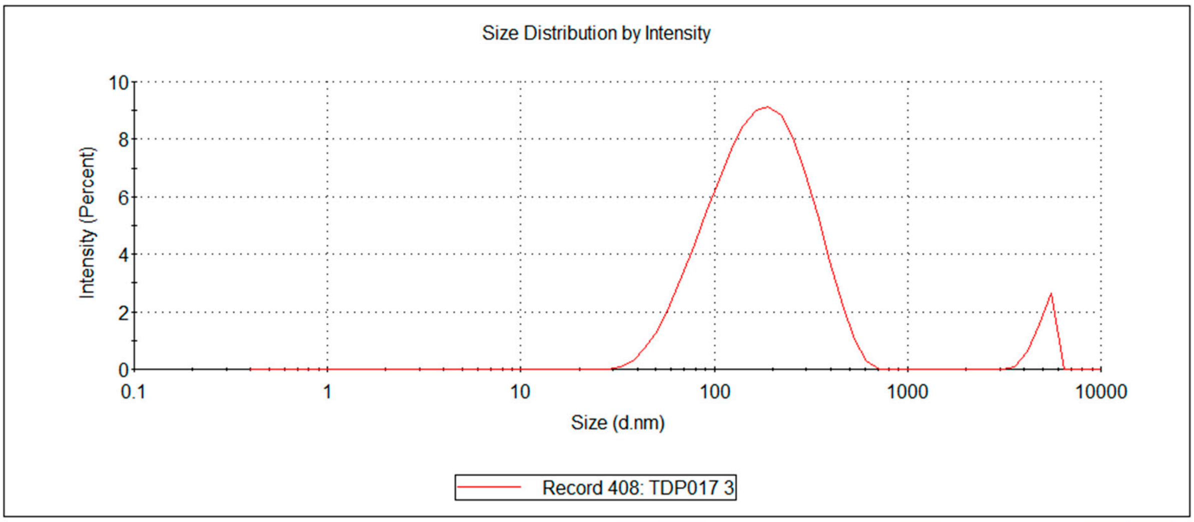

Figure S18. 0.5 g/L SDS, 5.0 V/V% EtOH

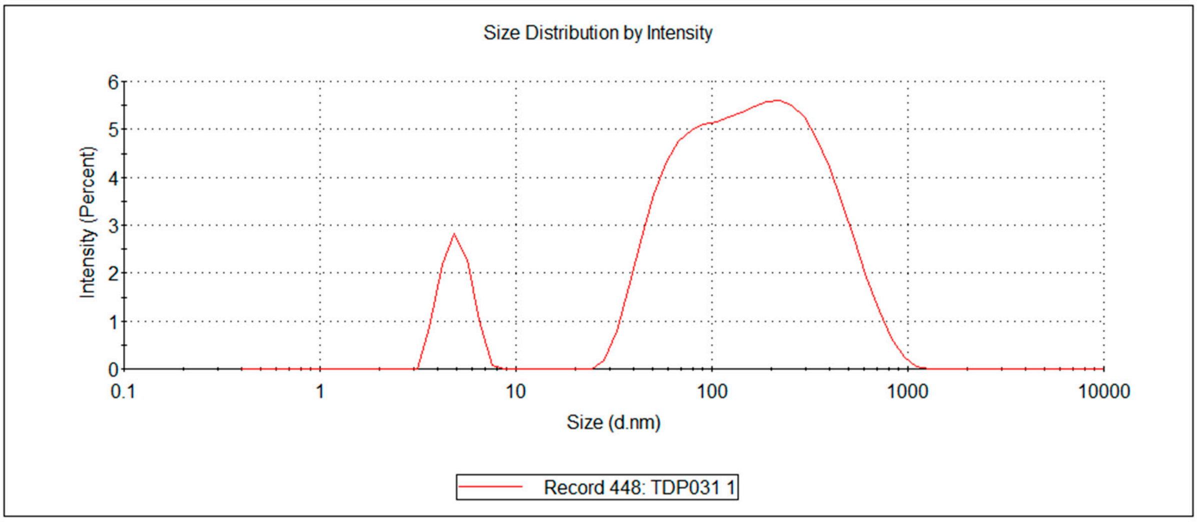

Figure S19. 1.0 g/L SDS, 5.0 V/V% EtOH

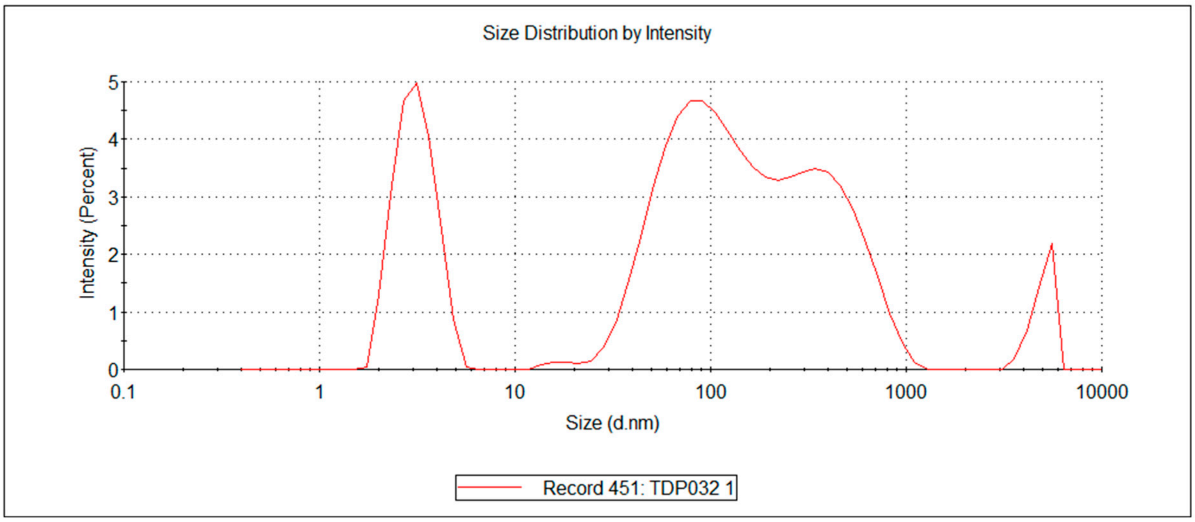

Figure S20. 2.5 g/L SDS, 5.0 V/V% EtOH

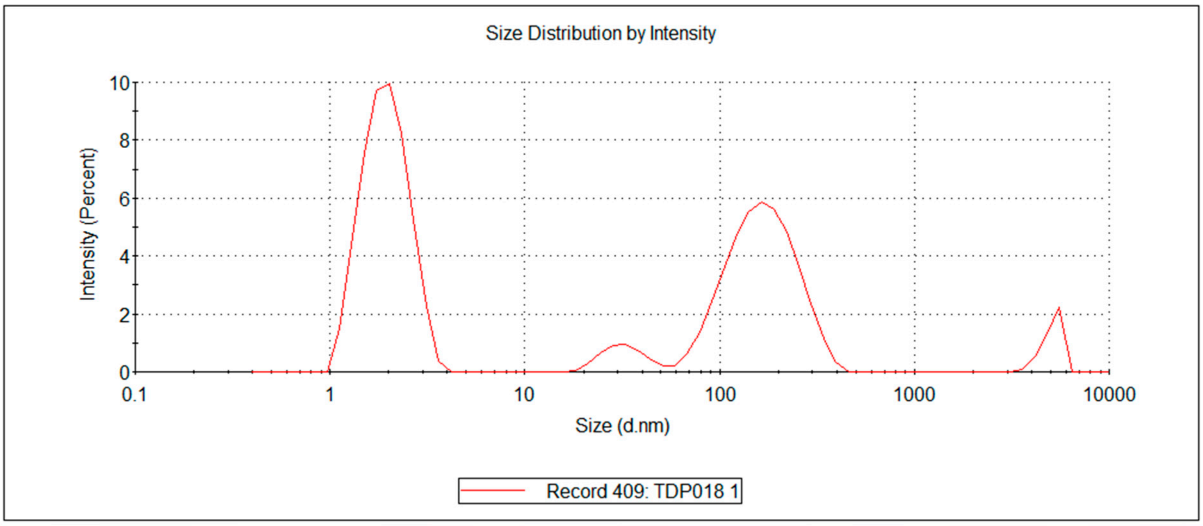

Figure S21. 5.0 g/L SDS, 5.0 V/V% EtOH

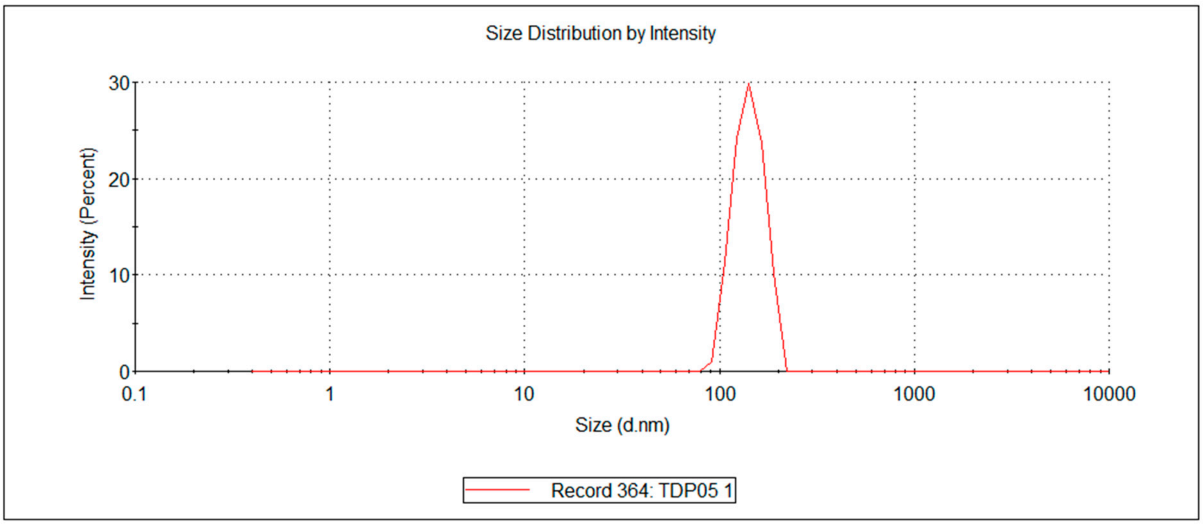

Figure S22. 0.5 g/L SDS, 10.0 V/V% EtOH

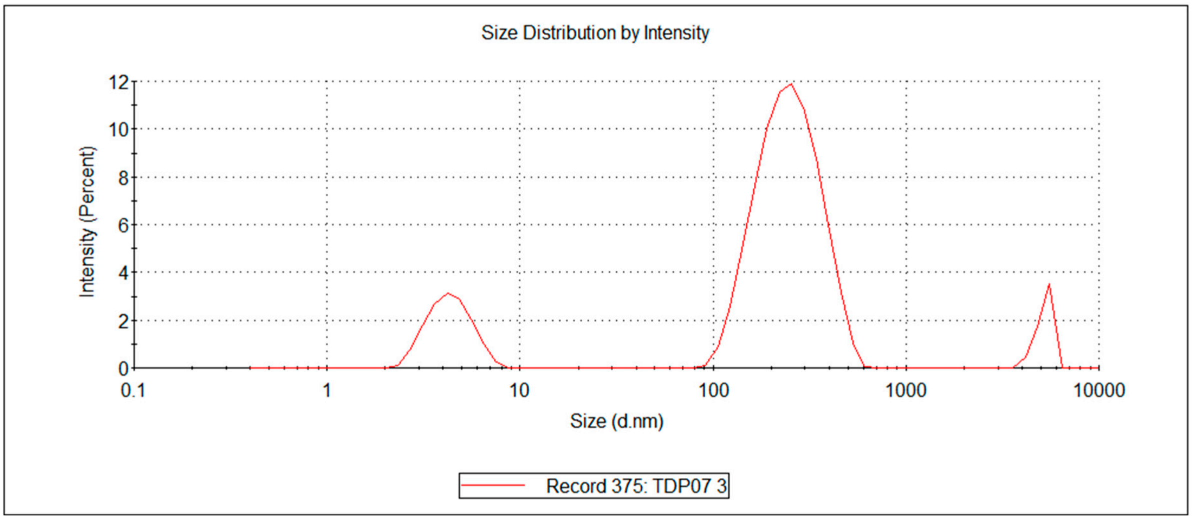

Figure S23. 1.0 g/L SDS, 10.0 V/V% EtOH

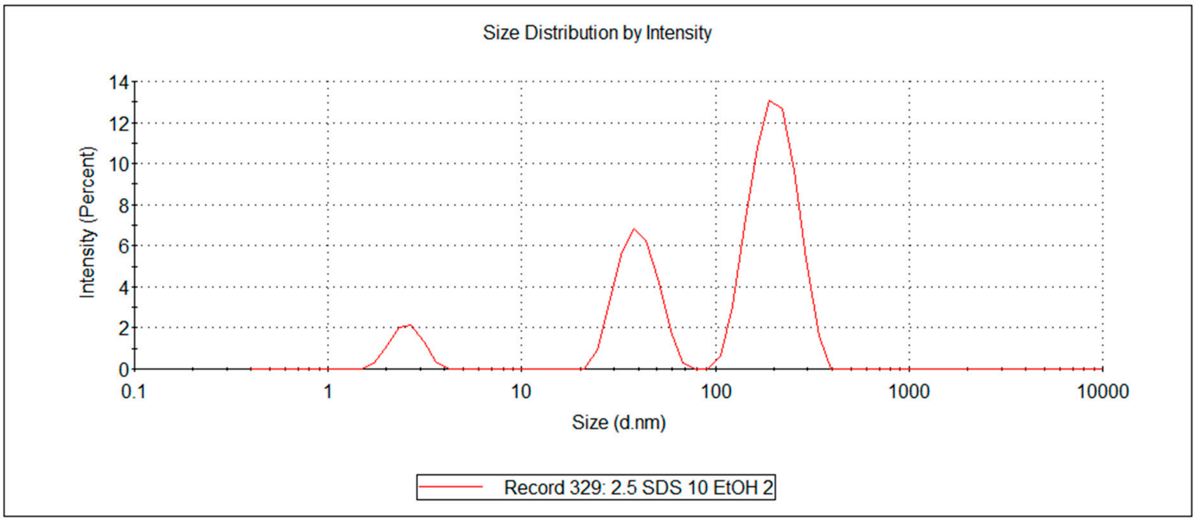

Figure S24. 2.5 g/L SDS, 10.0 V/V% EtOH

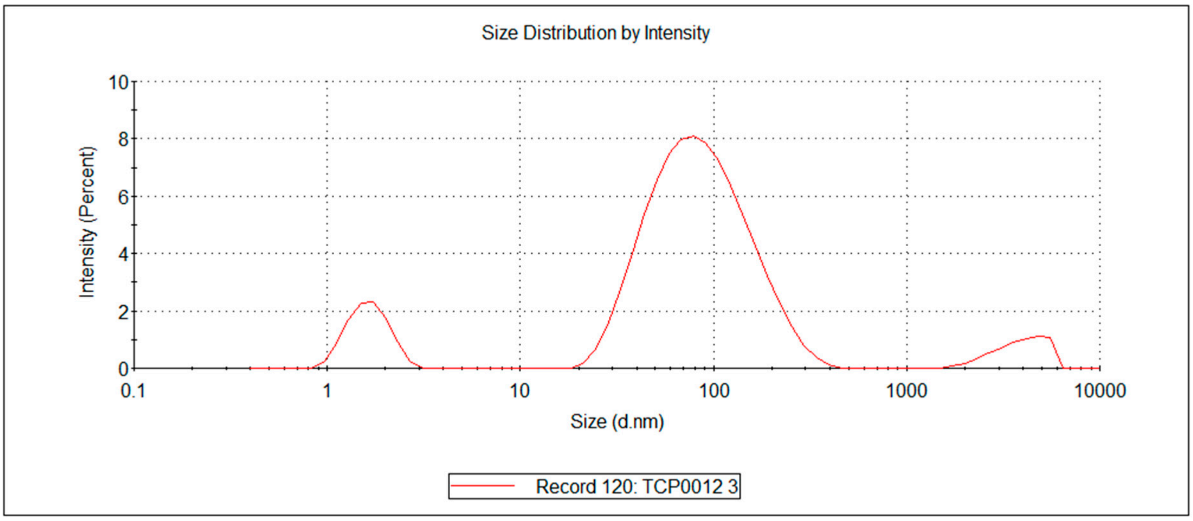

Figure S25. 5.0 g/L SDS, 10.0 V/V% EtOH

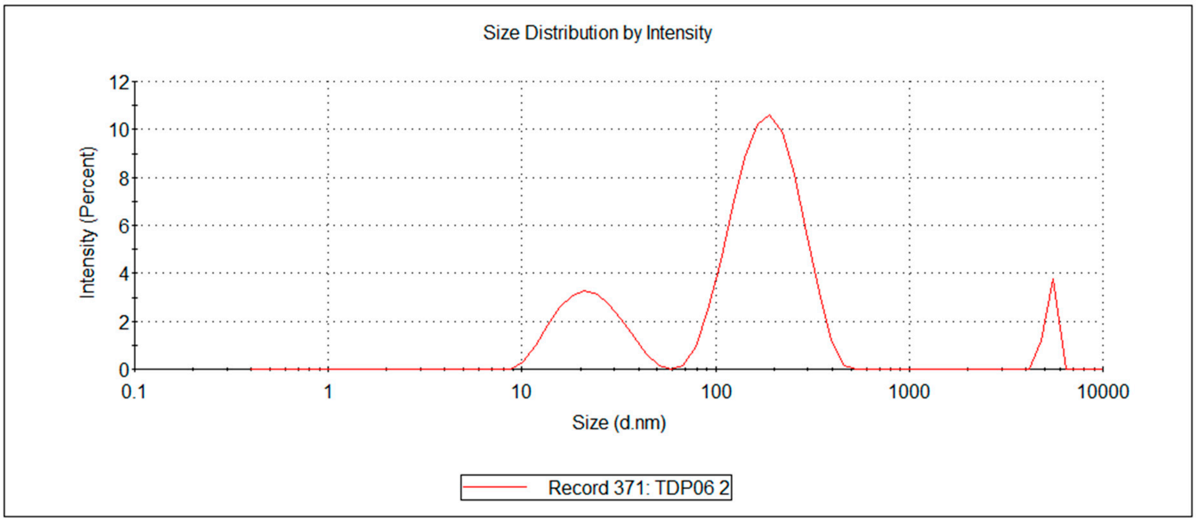

Figure S26. 0.5 g/L SDS, 20.0 V/V% EtOH

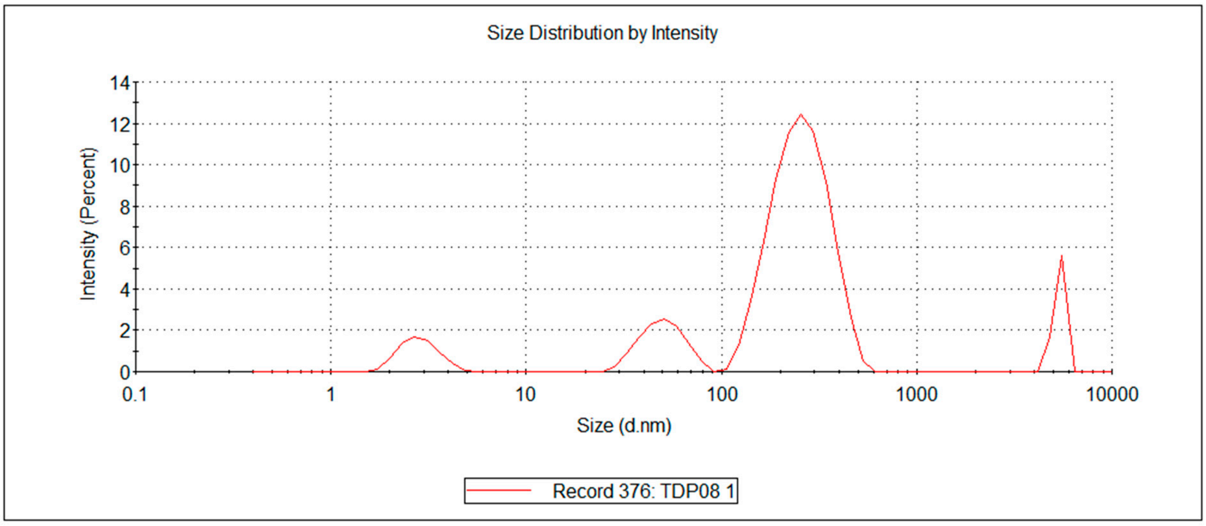

Figure S27. 1.0 g/L SDS, 20.0 V/V% EtOH

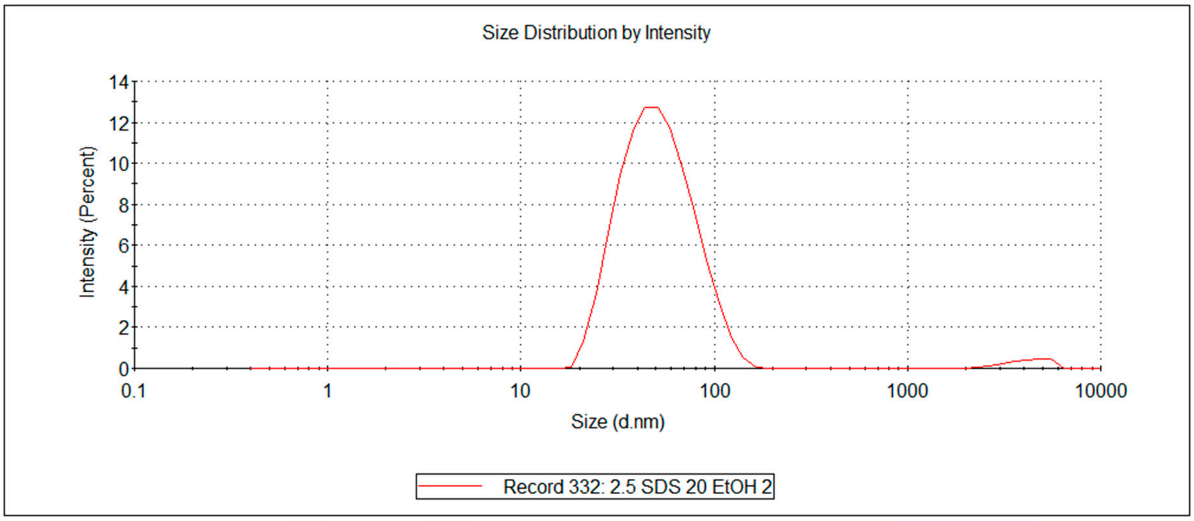

Figure S28. 2.5 g/L SDS, 20.0 V/V% EtOH

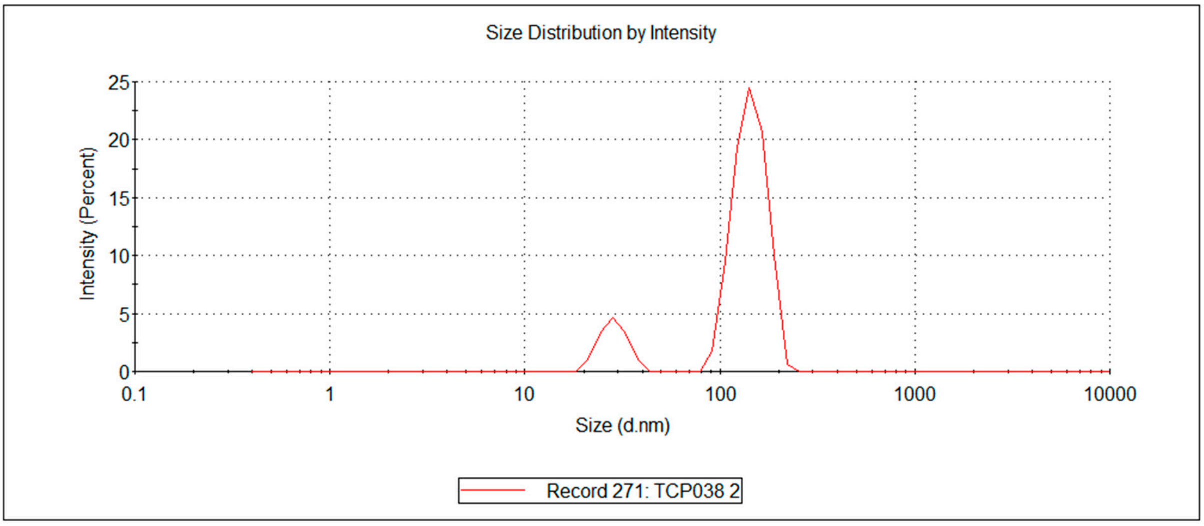

Figure S29. 5.0 g/L SDS, 20.0 V/V% EtOH

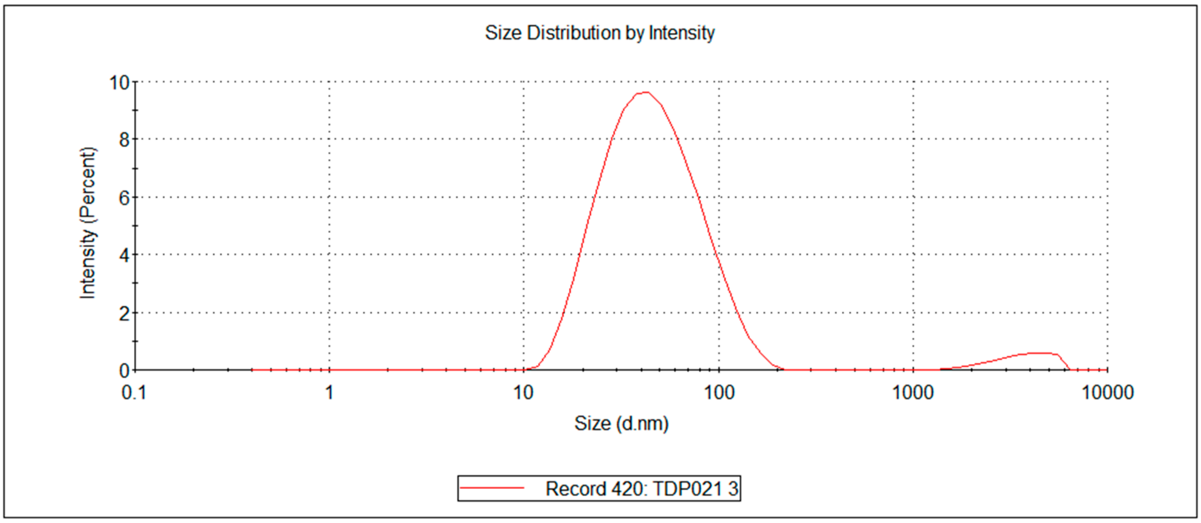

Figure S30. 0.5 g/L SDS, 5.0 V/V% *n*-propanol

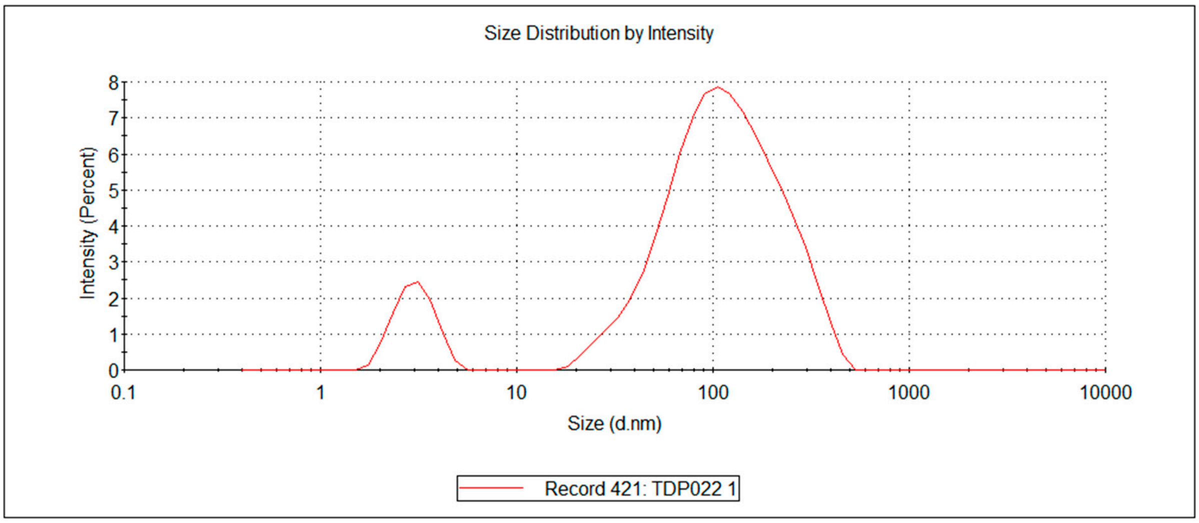

Figure S31. 1.0 g/L SDS, 5.0 V/V% *n*-propanol

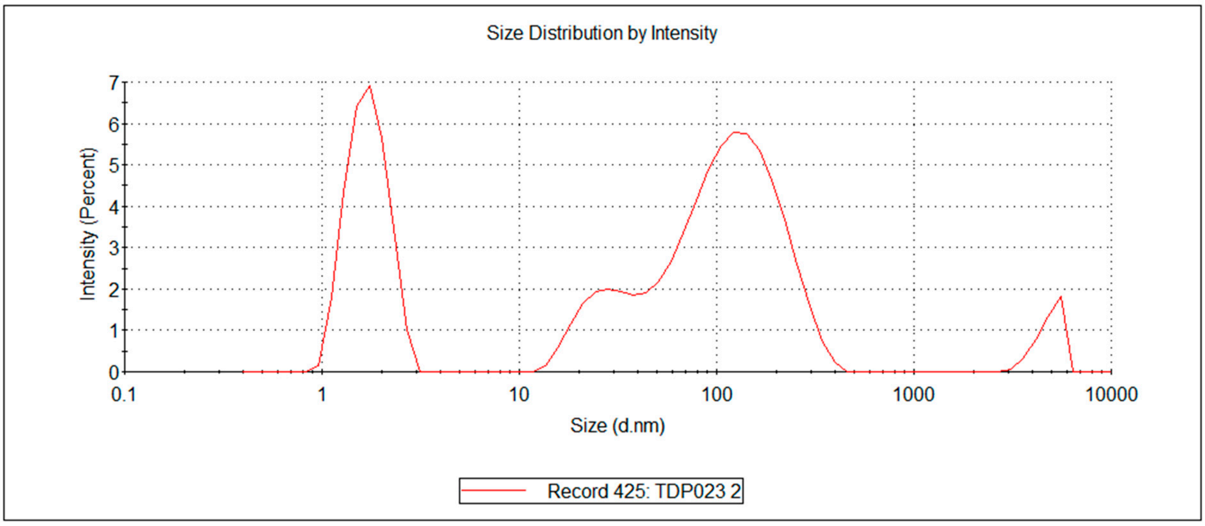

Figure S32. 2.5 g/L SDS, 5.0 V/V% *n*-propanol

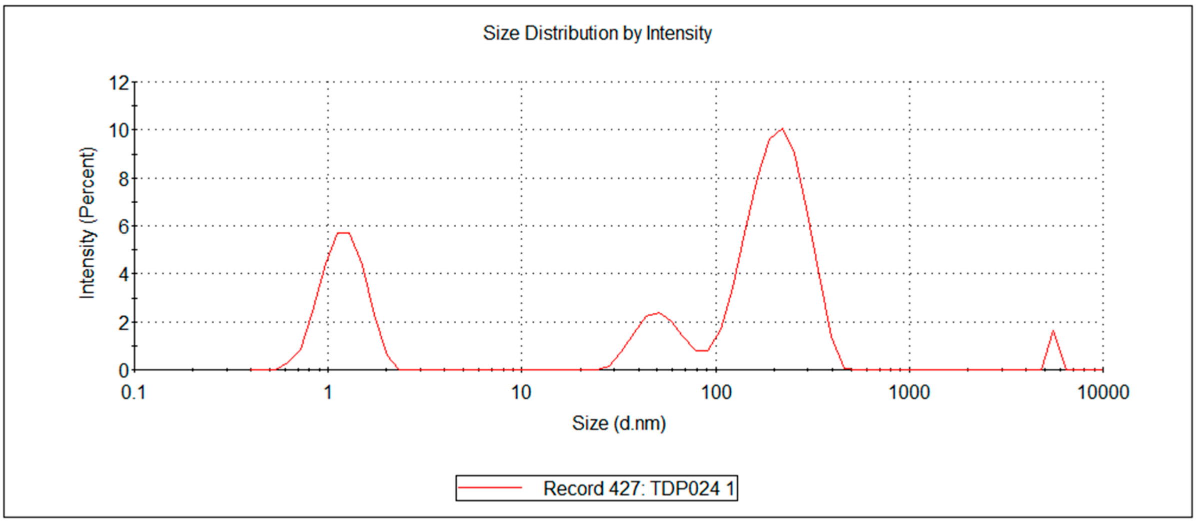

Figure S33. 5.0 g/L SDS, 5.0 V/V% *n*-propanol

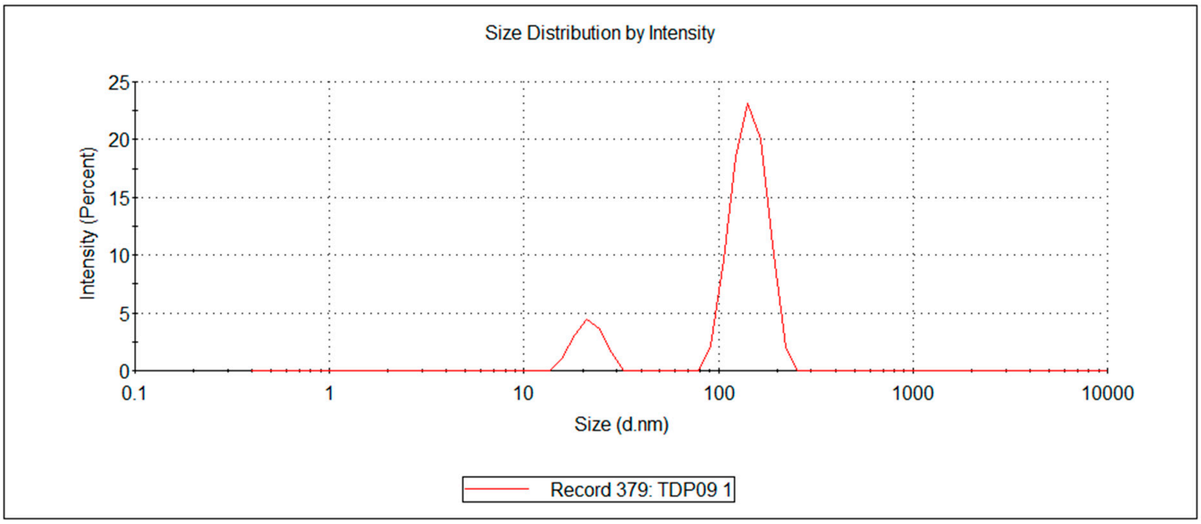

Figure S34. 0.5 g/L SDS, 10.0 V/V% *n*-propanol

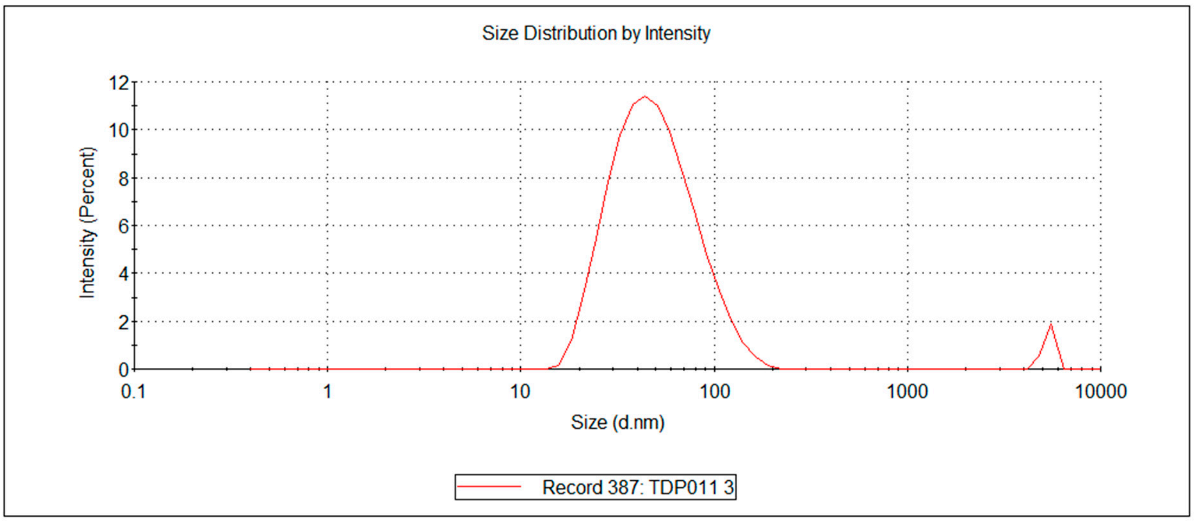

Figure S35. 1.0 g/L SDS, 10.0 V/V% *n*-propanol

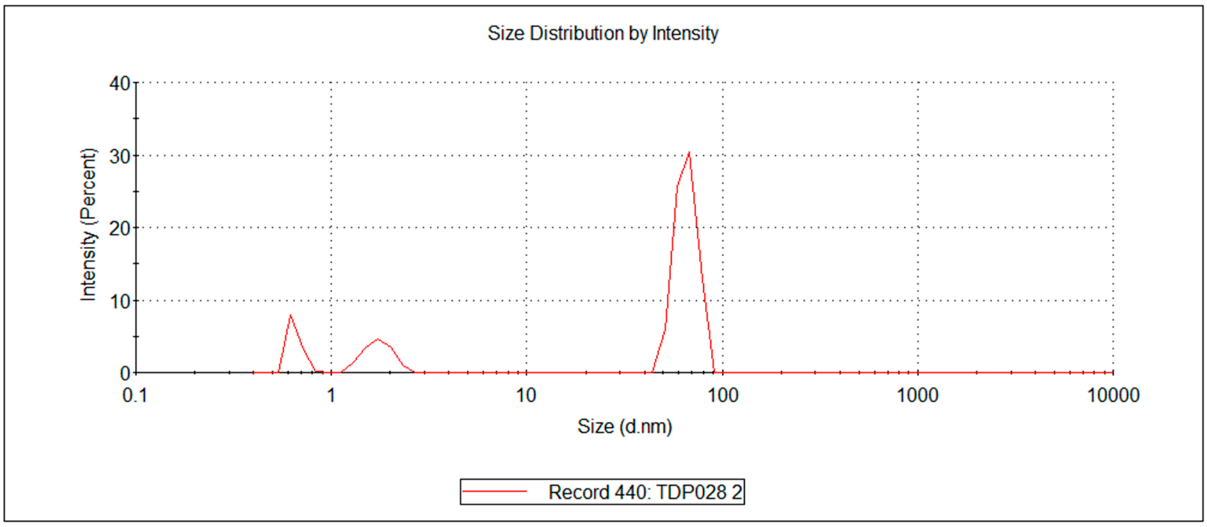

Figure S36. 2.5 g/L SDS, 10.0 V/V% *n*-propanol

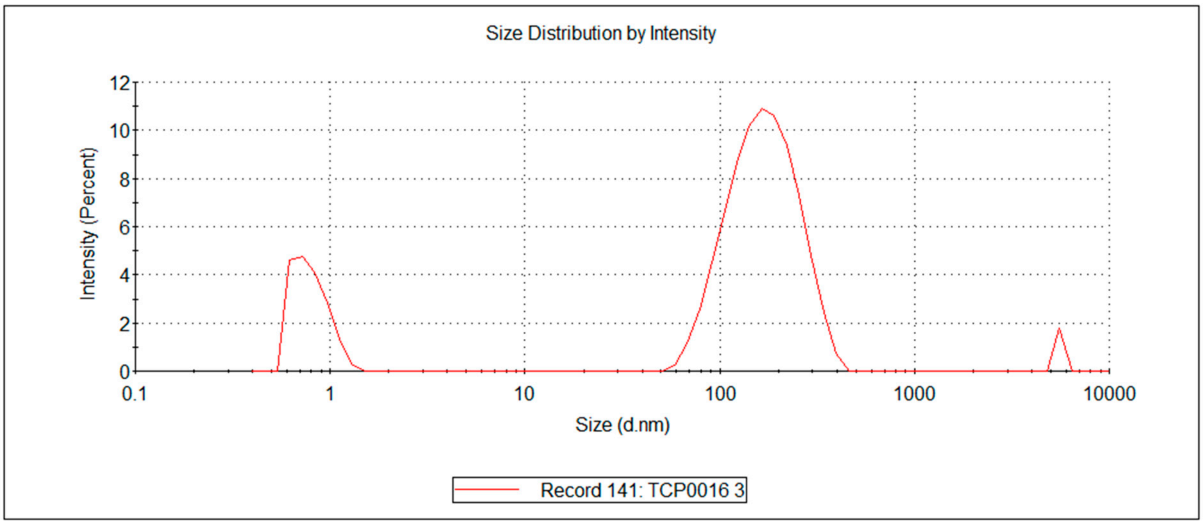

Figure S37. 5.0 g/L SDS, 10.0 V/V% *n*-propanol

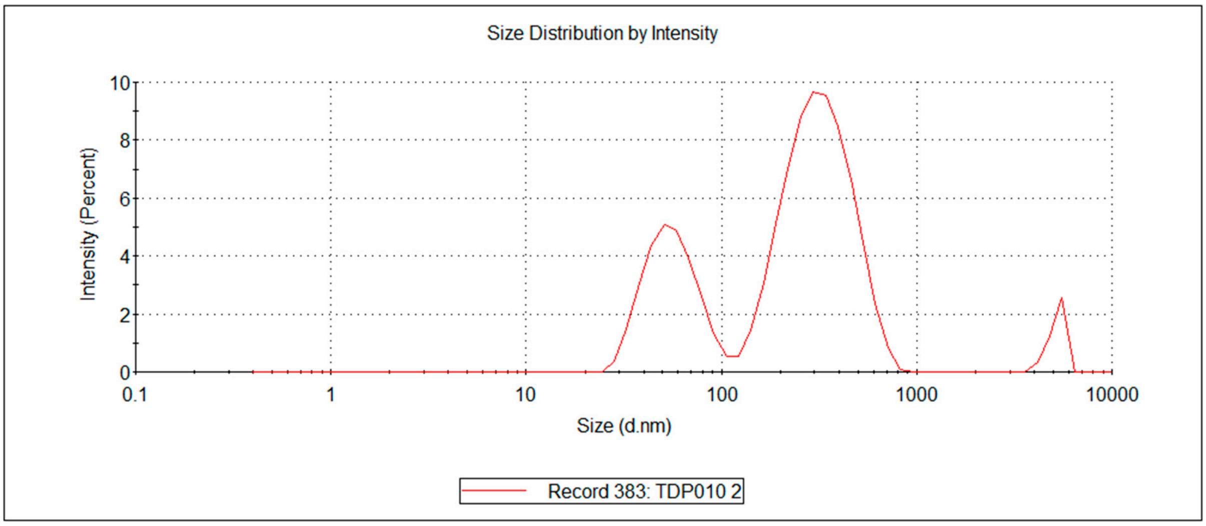

Figure S38. 0.5 g/L SDS, 20.0 V/V% *n*-propanol

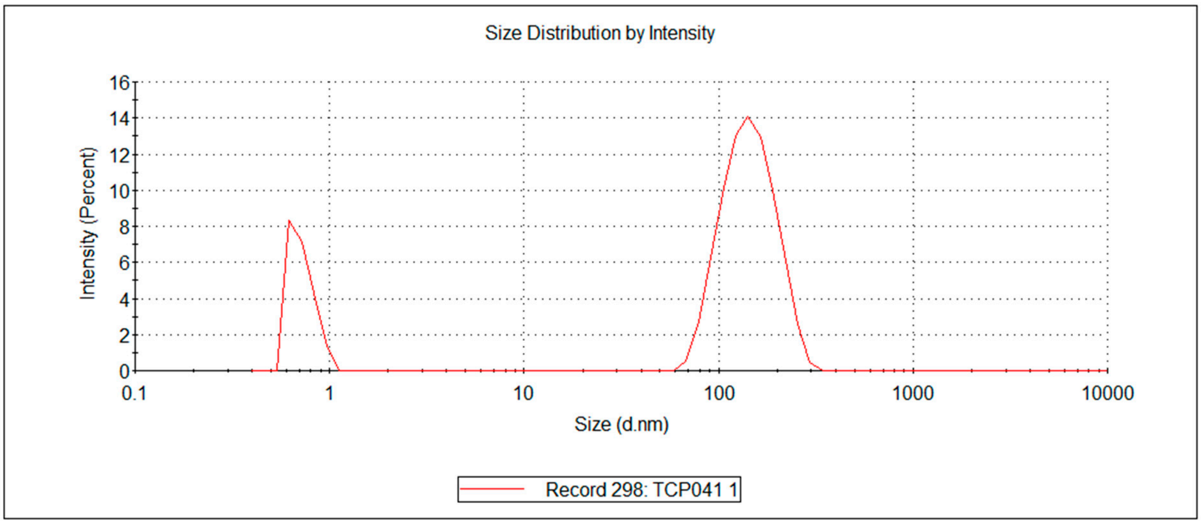

Figure S39. 1.0 g/L SDS, 20.0 V/V% *n*-propanol

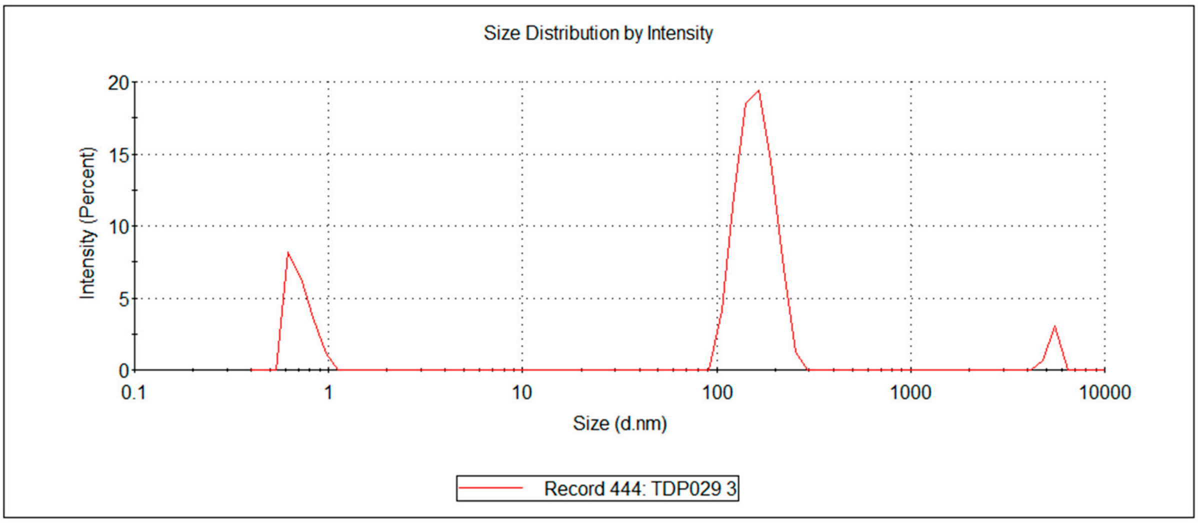

Figure S40. 2.5 g/L SDS, 20.0 V/V% *n*-propanol

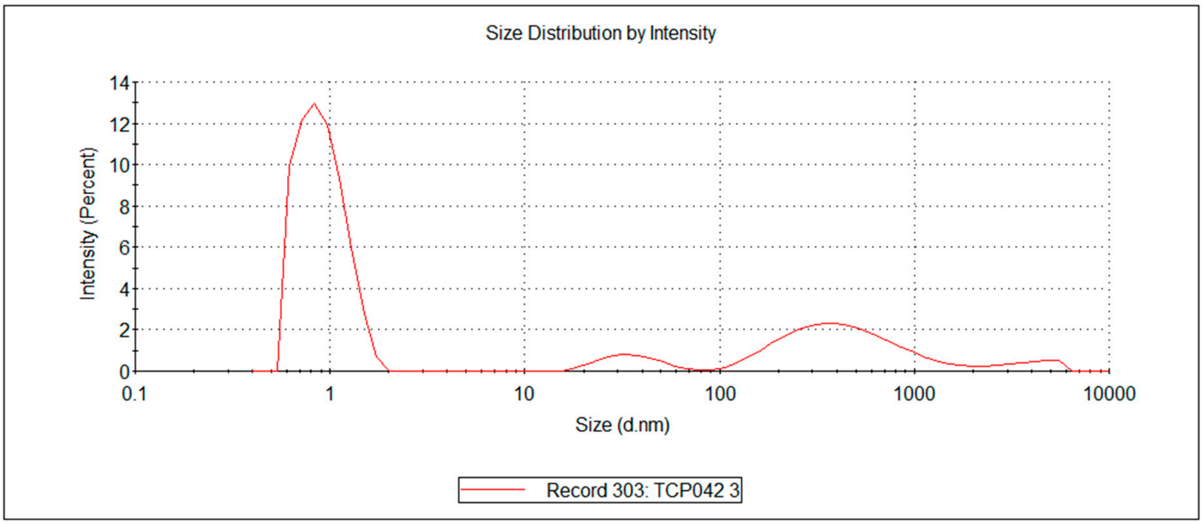

Figure S41. 5.0 g/L SDS, 20.0 V/V% *n*-propanol

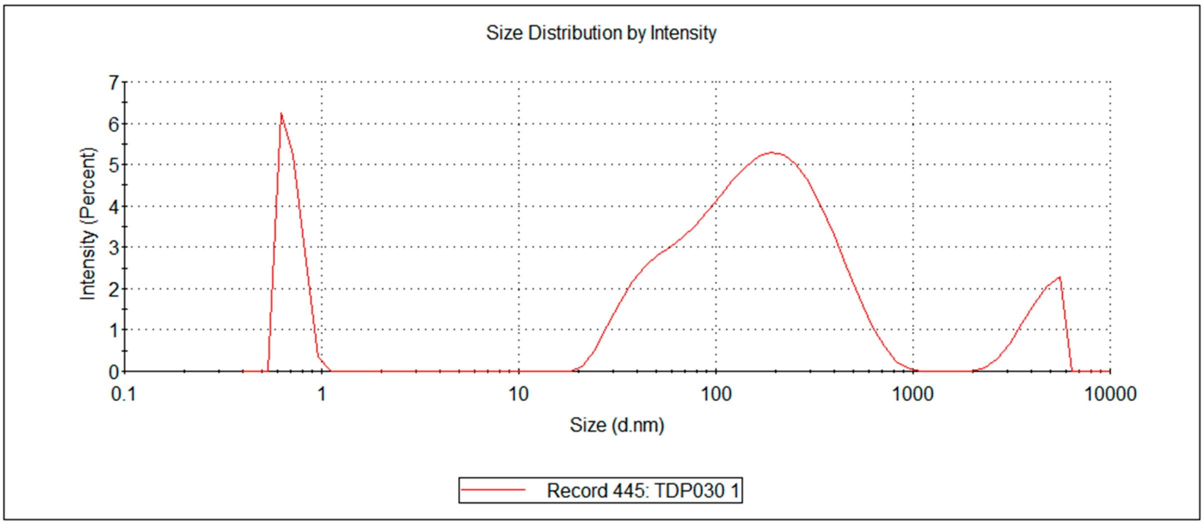

Figure S42. 0.1 g/L SDS, 5.0 V/V% *n*-butanol

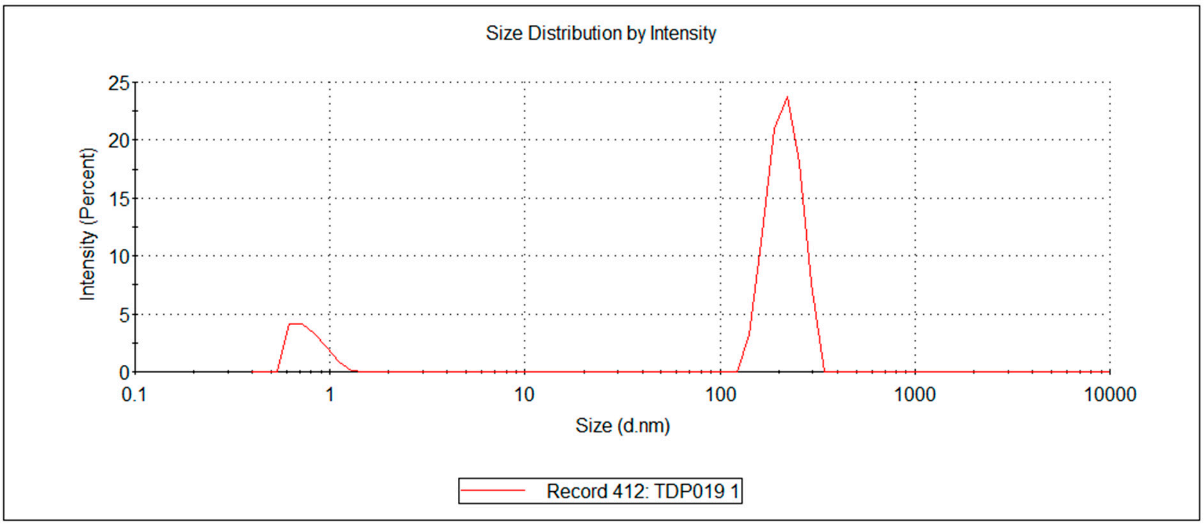

Figure S43. 0.5 g/L SDS, 5.0 V/V% *n*-butanol

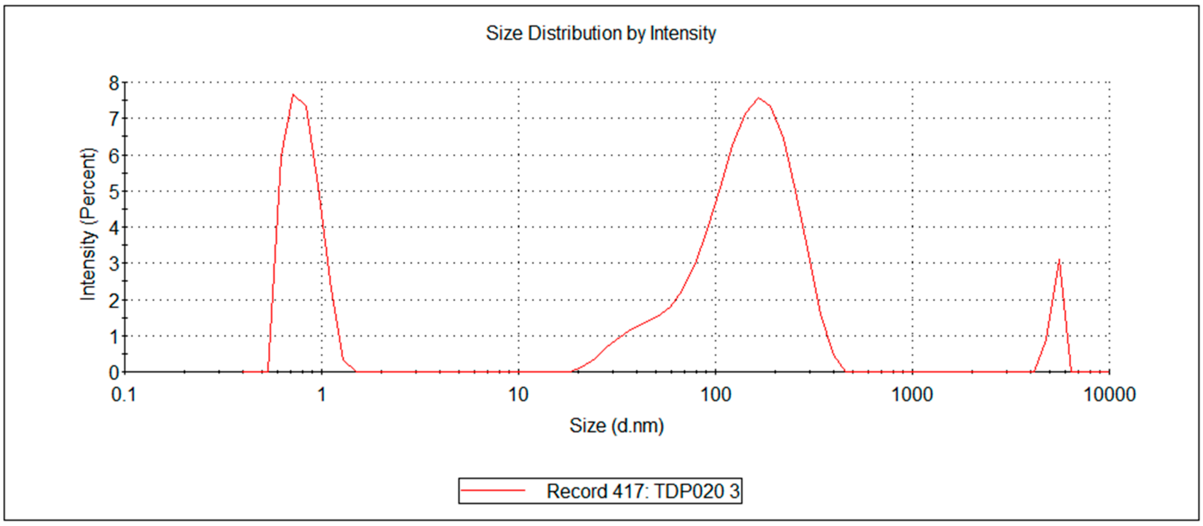

Figure S44. 1.0 g/L SDS, 5.0 V/V% *n*-butanol

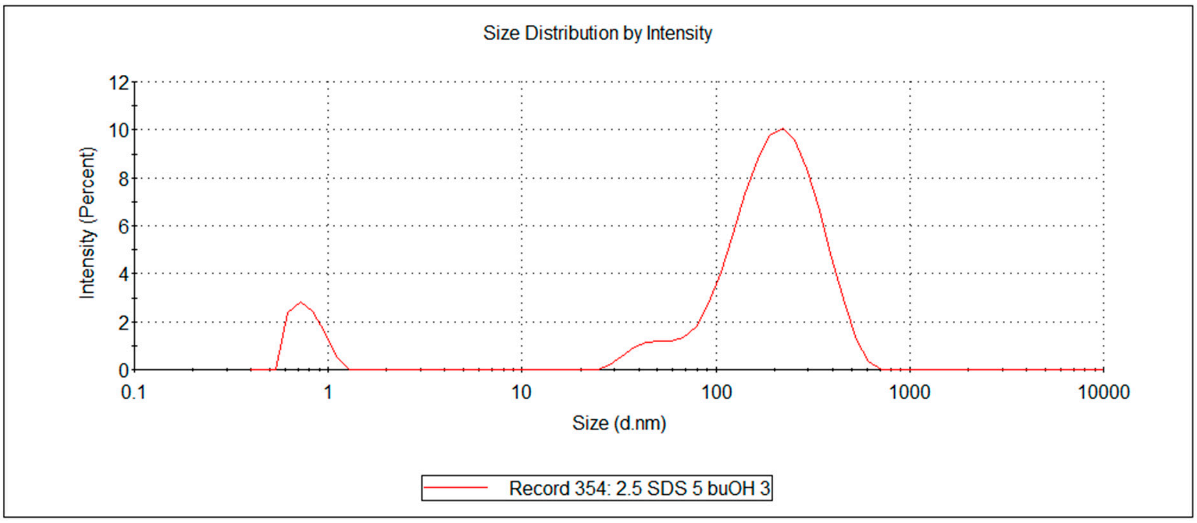

Figure S45. 2.5 g/L SDS, 5.0 V/V% *n*-butanol

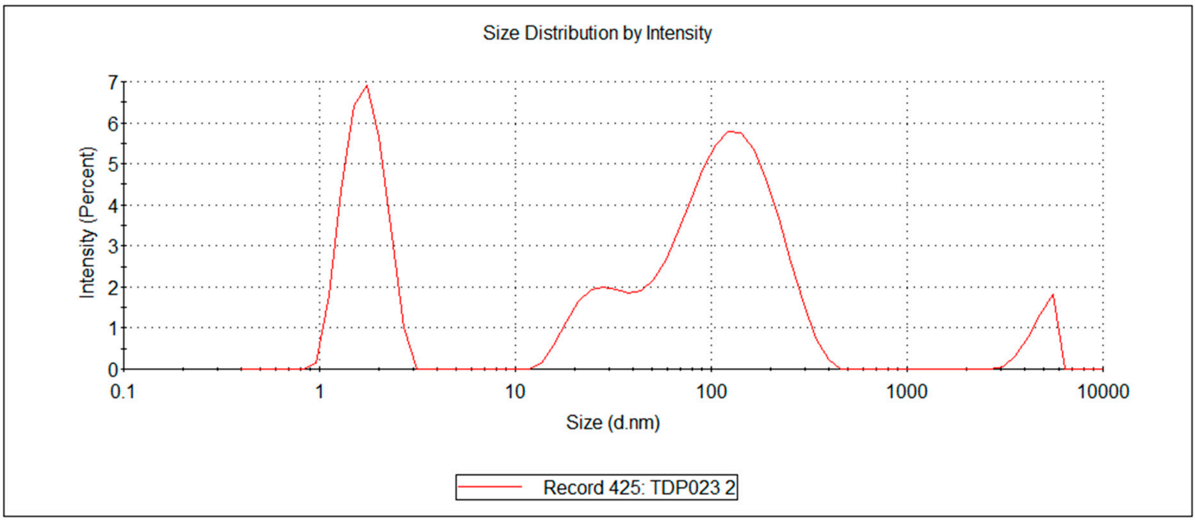

Figure S46. 5.0 g/L SDS, 5.0 V/V% *n*-butanol
